# Distinct evolutionary dynamics of horizontal gene transfer in drug resistant and virulent clones of *Klebsiella pneumoniae*

**DOI:** 10.1371/journal.pgen.1008114

**Published:** 2019-04-15

**Authors:** Kelly L. Wyres, Ryan R. Wick, Louise M. Judd, Roni Froumine, Alex Tokolyi, Claire L. Gorrie, Margaret M. C. Lam, Sebastián Duchêne, Adam Jenney, Kathryn E. Holt

**Affiliations:** 1 Department of Infectious Diseases, Monash University, Melbourne, Victoria, Australia; 2 Department of Biochemistry and Molecular Biology, Bio21 Molecular Science and Biotechnology Institute, University of Melbourne, Parkville, Victoria, Australia; 3 Department of Infectious Diseases and Microbiology Unit, The Alfred Hospital, Melbourne, Victoria, Australia; 4 Microbiological Diagnostic Unit Public Health Laboratory, Department of Microbiology and Immunology, University of Melbourne, Peter Doherty Institute for Infection and Immunity, Melbourne, Victoria, Australia; 5 London School of Hygiene and Tropical Medicine, London, United Kingdom; Uppsala University, SWEDEN

## Abstract

*Klebsiella pneumoniae* has emerged as an important cause of two distinct public health threats: multi-drug resistant (MDR) healthcare-associated infections and drug susceptible community-acquired invasive infections. These pathotypes are generally associated with two distinct subsets of *K*. *pneumoniae* lineages or ‘clones’ that are distinguished by the presence of acquired resistance genes and several key virulence loci. Genomic evolutionary analyses of the most notorious MDR and invasive community-associated (‘hypervirulent’) clones indicate differences in terms of chromosomal recombination dynamics and capsule polysaccharide diversity, but it remains unclear if these differences represent generalised trends. Here we leverage a collection of >2200 *K*. *pneumoniae* genomes to identify 28 common clones (n ≥ 10 genomes each), and perform the first genomic evolutionary comparison. Eight MDR and 6 hypervirulent clones were identified on the basis of acquired resistance and virulence gene prevalence. Chromosomal recombination, surface polysaccharide locus diversity, pan-genome, plasmid and phage dynamics were characterised and compared. The data showed that MDR clones were highly diverse, with frequent chromosomal recombination generating extensive surface polysaccharide locus diversity. Additional pan-genome diversity was driven by frequent acquisition/loss of both plasmids and phage. In contrast, chromosomal recombination was rare in the hypervirulent clones, which also showed a significant reduction in pan-genome diversity, largely driven by a reduction in plasmid diversity. Hence the data indicate that hypervirulent clones may be subject to some sort of constraint for horizontal gene transfer that does not apply to the MDR clones. Our findings are relevant for understanding the risk of emergence of individual *K*. *pneumoniae* strains carrying both virulence and acquired resistance genes, which have been increasingly reported and cause highly virulent infections that are extremely difficult to treat. Specifically, our data indicate that MDR clones pose the greatest risk, because they are more likely to acquire virulence genes than hypervirulent clones are to acquire resistance genes.

## Author summary

*Klebsiellla pneumoniae* is classified by the World Health Organization as a priority drug-resistant organism because it causes a significant burden of hospital infections that are extremely difficult to treat. However, outside of the hospital setting this bacterium is also an important cause of severe drug-susceptible infections. Until recently these two infection types were associated with distinct subsets of the *K*. *pneumoniae* population harbouring high prevalence of drug-resistance and virulence genes, respectively. However, there are now increasing reports of highly-virulent and difficult-to-treat *K*. *pneumoniae* strains that carry both resistance and virulence genes. In this study, we used genomic analyses to characterise and compare the evolutionary histories of drug-resistant and virulent *K*. *pneumoniae*. We show that the former are highly diverse, frequently acquiring novel genes through the processes of chromosomal recombination, plasmid and bacteriophage acquisition. In comparison, the latter show considerably lower gene content diversity, suggesting that they may be subject to some sort of limitation for gene acquisition. Consequently, we predict that drug-resistant *K*. *pneumoniae* are more likely to acquire virulence genes than virulent *K*. *pneumoniae* are to acquire drug-resistance genes.

## Introduction

*Klebsiella pneumoniae* is most well known as an opportunistic hospital pathogen for which multi-drug resistance (MDR) is a major global public health concern [1]. However, this bacterium has also emerged as an important cause of community acquired invasive disease, often manifesting as liver abscess with bacteraemia and usually susceptible to antimicrobials [2]. The *K*. *pneumoniae* population is extemerly diverse, comprising 100s of independent phylogenetic lineages or ‘clones’ that differ from each other by ~0.5% nucletide divergence [1]. The majority of MDR hospital outbreaks are caused by a small subset of *K*. *pneumoniae* clones with a high prevalence of acquired antimicrobial resistance (AMR) genes, while the majority of community-acquired invasive infections are caused by ‘hypervirulent’ clones that rarely harbour acquired AMR genes but have high prevalence of key virulence loci [1–3].

In *K*. *pneumoniae* MDR evolution is largely driven through acquisition of AMR genes on diverse mobilisable plasmids [4] which are particularly prevalent among the global hospital outbreak clones [5]; e.g. clonal group (CG) 258 which is implicated in global spread of the *K*. *pneumoniae* carbapenemases [6]. *K*. *pneumoniae* pathogenicity is driven by a wide array of interacting factors [7–9] that are present in all strains, including the type III fimbriae (*mrk*) and the surface polysaccharides (capsule and lipopolysaccharide (LPS)) [9,10] which exhibit antigenic variation between strains. The majority of hypervirulent *K*. *pneumoniae*, distinguished clinically as causing invasive infections even outside the hospital setting [11], are associated with just two [3,12] of the >130 predicted capsular serotypes [13], K1 and K2, that are considered particularly antiphagocytic and serum resistant [12,14]. Hypervirulent *K*. *pneumoniae* are also associated with high prevalence of several other key virulence factors; the *rmpA*/*rmpA2* genes that upregulate capsule expression to generate hypermucoidy [15,16]; the colibactin genotoxin that induces eukaryotic cell death and promotes invasion to the blood from the intestines [17,18]; and the yersiniabactin, aerobactin and salmochelin siderophores that promote survival in the blood by enhancing iron sequestration [8,19–21].

Yersiniabactin synthesis is encoded by the *ybt* locus, which is usually mobilised by an integrative, conjugative element known as ICE*Kp*. It is present in ~40% of the general *K*. *pneumoniae* population and seems to be frequently acquired and lost from MDR clones [22]. Fourteen distinct *ybt*+ICE*Kp* variants are recognised, one of which also carries the colibactin synthesis locus (*clb*) [22]. In contrast, the salmochelin (*iro*), aerobactin (*iuc*) and *rmpA*/*rmpA2* loci are usually co-located on a virulence plasmid [23,24]. These loci are much less common in the *K*. *pneumoniae* population (<10% prevalence each) and until recently were rarely reported among MDR strains [1,2].

The reasons for the apparent separation of MDR and hypervirulence are unclear but there are growing reports of convergence from both directions, i.e. hypervirulent strains gaining MDR plasmids [25–30] and MDR strains gaining a virulence plasmid plus/minus an ICE*Kp* [27,31,32]. Most such reports are sporadic, but in 2017 Gu and colleagues described a fatal outbreak of MDR, carbapenem-resistant *K*. *pneumoniae* belonging to CG258 that had acquired ICE*Kp* in the chromosome plus *iuc* and *rmpA2* on a virulence plasmid [31]. The report fuelled growing fears of an impending public health disaster in which highly virulent MDR strains may be able to spread in the community, causing dangerous infections that are extremely difficult to treat [33]. However, there remain significant knowledge gaps about *K*. *pneumoniae* evolution that limit our ability to understand the severity of this public health threat, and to evaluate the relative risks of convergence events.

The vast majority of clonal evolutionary analyses have focussed on a single MDR clone, CG258, revealing an evolutionary history peppered by chromosomal recombination events, extensive capsule locus diversity and acquisition of diverse AMR genes on diverse plasmids (summarised in [5]). Few studies have explored the genomic evolution of other clones, but our recent study of hypervirulent CG23 highlighted intriguing differences; i) chromosomal recombination and plasmid acquisition were comparatively rare and; ii) a single capsule locus and the virulence plasmid were both maintained for >100 years [28].

Here we report the first comprehensive comparison of genome evolutionary dynamics in multiple *K*. *pneumoniae* clones. We leveraged a curated collection of >2200 *K*. *pneumoniae* genomes to identify 28 common clones with at least 10 genomes in each. We characterised each clone in terms of its resistance and virulence gene content, and surface polysaccharide diversity. We also performed chromosomal recombination and pan-genome analyses, revealing key differences in horizontal gene transfer dynamics.

## Results and discussion

### Definition of hypervirulent and MDR clones

**Fig 1:**
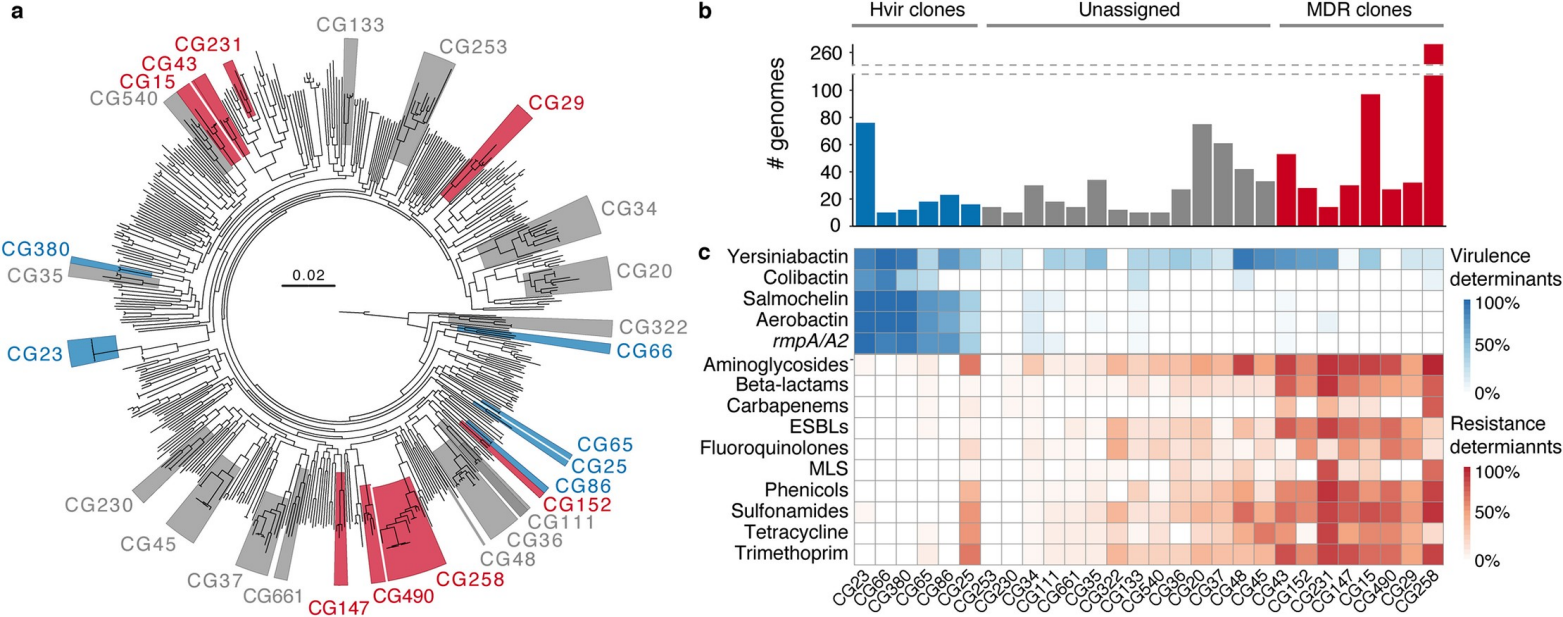
Definition of *K*. *pneumoniae* clones investigated in this study. **a)** Phylogenetic tree inferred using maximum likelihood for *K*. *pneumoniae* genomes selected from our curated collection to represent the 509 distinct 7-gene chromosomal multi-locus sequence types. Phylogenetic clusters (monophyletic groups) were defined using patristic distance (cut-off = 0.04). Clusters corresponding to clones included in comparative analyses are marked; blue, hypervirulent; grey, unassigned; red, multi-drug resistant. **b)** Total number of genomes included in comparative analyses, coloured by clone type as above. Note that sample sizes exceed the number of isolates shown in the tree for the corresponding clones. **c)** Distribution of virulence and resistance determinants by clone. Intensity of box shading indicates the proportion of genomes harbouring the key virulence loci (blue) or acquired genes conferring resistance to different classes of antimicrobials (red), as per inset legends. Hypervirulent (Hvir) clones were defined by hierarchical clustering of virulence locus data. Multi-drug resistant (MDR) clones were defined by hierarchical clustering of resistance data. AMR, antimicrobial resistance; *rmpA*/*A2*, regulators of mucoid phenotype; ESBLs, extended spectrum beta-lactams; MLS, macrolide, lincosamide and streptogramin B antibiotics.

**Fig 2:**
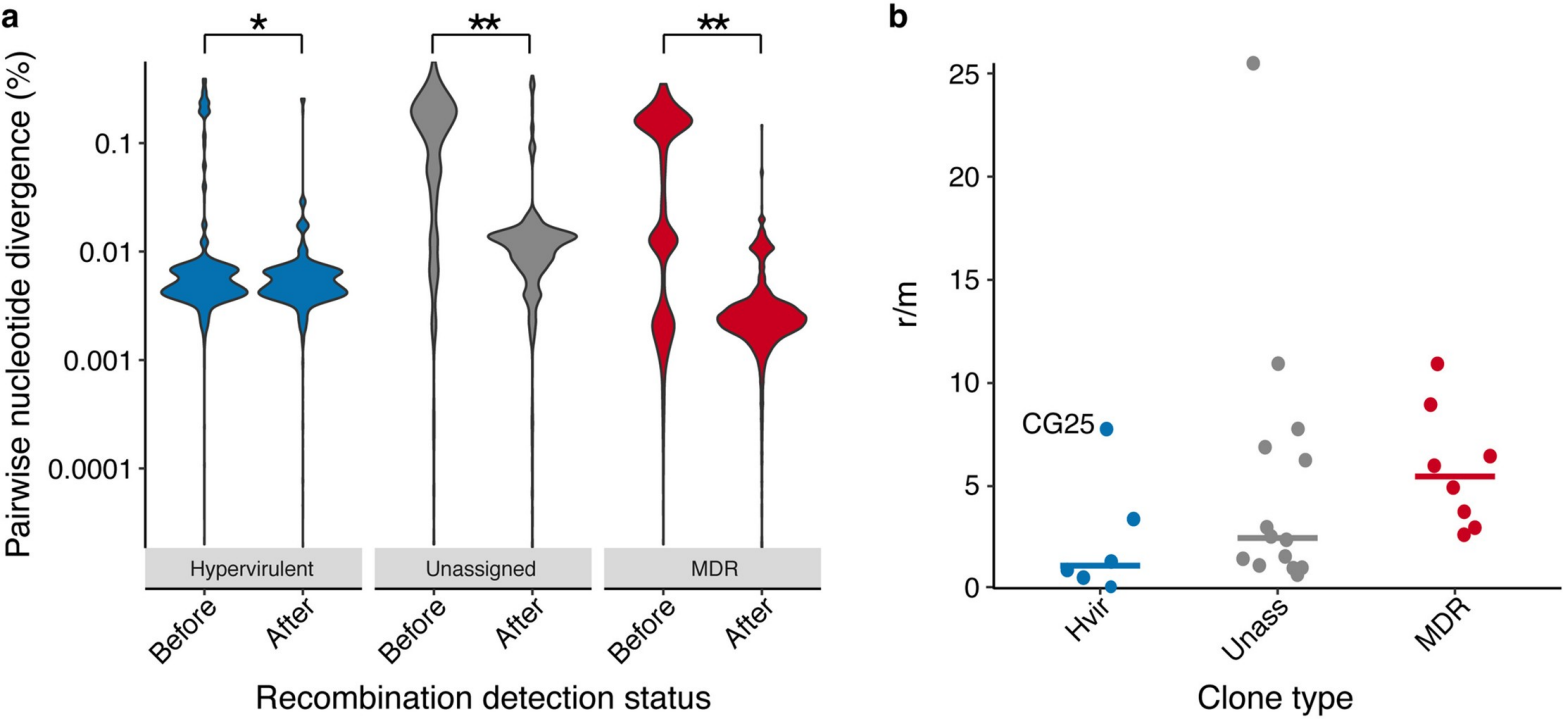
Nucleotide divergence and recombination dynamics. **a)** Violin plots showing distributions of pairwise nucleotide divergences grouped by clone type. Data points represent comparisons between pairs of genomes within clones. Pairwise values were clalculated before and after removal of recombinant sequence regions identified by Gubbins [35]. *, p < 0.001; **, p < 1x10^-15^. **b)** Scatter plot showing the ratio of single nucleotide polymorphisms introduced by recombination vs mutation (r/m) for each clone grouped by clone type (n = 6, 14 and 8 for the hypervirulent, unassigned and MDR groups, respectively). Bars indicate median values.

A total of 28 common *K*. *pneumoniae* clones were identified from our collection of 2265 *K*. *pneumoniae* genomes as described in **Methods** (total 1092 genomes, 10–266 genomes per clone, see **Fig 1A and 1B, S1 and S2 Tables**). The majority (87.6%) represented human clinical or carriage isolates from a range of geographies and years, such that ≥3 geographic continents and ≥6 years of time were represented within each clone **(S1 Fig)**. Unless otherwise stated, there were no statistically significant associations between features of the genome sample for each clone (including sample size, median evolutionary divergence, or geographic diversity) and results of the genomic analyses discussed hereafter (see **S1 Text, S3 Table**, and **Fig 2A** as discussed below).

For each clone, the proportion of genomes harbouring the key virulence and AMR loci were calculated (**Fig 1C**). Hierarchical clustering of the virulence locus data were used to define a group of 6 hypervirulent clones including the previously described CG23, CG86 and CG65 [2], each of which harboured the virulence plasmid-associated genes *iuc*, *iro* and/or *rmpA/rmpA2* at high frequency (31–100%). Hierarchical clustering of the AMR data were used to define a group of 8 MDR clones, including the outbreak associated CG258, CG15 and CG147 [5], each with a high frequency (≥56%) of genomes encoding acquired resistance determinants for ≥3 drug classes (in addition to ampicillin to which all *K*. *pneumoniae* share intrinsic resistance via the chromosomally encoded SHV-1 beta-lactamase, equivalent to the standard definition of MDR as described in [34]). As expected, AMR genes were rare among the hypervirulent clones, with the exception of CG25 in which 11 of 16 genomes harboured ≥4 acquired AMR genes. The *iuc*, *iro* and *rmpA*/*rmpA2* loci were rare (<12% frequency) among the MDR clones and those not assigned to either group (‘unassigned’ clones); however, the *ybt* locus was frequently identified across the spectrum of clones as has been reported previously [22].

This asymmetric distribution of virulence plasmid-associated virulence loci (*iro*, *iuc* and *rmpA/2*) with AMR determinants is in accordance with general trends seen across the wider *K*. *pneumoniae* population wherein the presence of the virulence plasmid (with or without ICE*Kp*) is negatively associated with the presence of acquired AMR genes: Among a broad, representative genome sample (n = 1124, see **Methods**) the majority (n = 77/88) of genomes harbouring the virulence plasmid contained 0–1 acquired AMR genes, while the distributions were much broader for genomes without the virulence plasmid plus/minus ICE*Kp* (median 1, interquartile range (IQR) 0–9, p < 1x10^-10^ for both pairwise Wilcoxon Rank Sum tests). Among the genomes without the virulence plasmid the distribution of acquired AMR genes was slightly shifted towards higher numbers in genomes harbouring ICE*Kp* (median 1 vs 1, IQR 0–8 vs 0–10, p < 1x10^-8^; see **S2 Fig**).

### The capsule and LPS synthesis loci are recombination hot-spots in MDR but not hypervirulent clones

We used Gubbins [35] to identify putative chromosomal recombination imports within each clone and calculated nucleotide divergence for all pairs of genomes within clones before and after the removal of recombinant sequence regions (**S3 Fig**). Median pairwise nucleotide divergence ranged from 0.003% to 0.355% prior to recombination detection and from 0.002% to 0.251% after recombination detection. The data indicated that recombination has significantly influenced within-clone nucleotide diversity for all clone types (**Fig 2A**), with particularly strong influence on the unassigned and MDR clones compared to the hypervirulent clones (pairwise Wilcoxon Rank Sum tests for difference in nucleotide diversity after recombination removal; MDR and unassigned, p < 1x10^-15^; hypervirulent, p = 0.0004). Notably, within-clone nucleotide divergence differed by clone type, both prior and following the removal of recombinant regions (Kruskal-Wallis test, p < 1x10^-15^). Importantly, these data showed that our sample captured less nucleotide divergence due to accumulation and vertical inheritance of substitution mutations among the MDR than hypervirulent clones (median pairwise divergence after removal of recombinant regions; 0.003% vs 0.005%, p < 1x10^-15^ by Wilcoxon Rank Sum test). Nucleotide divergence through ancestral descent is generally considered to be correlated with evolutionary time, and these observations are consistent with recent molecular dating analyses estimating that MDR CG258 emerged in the mid 1980s [36] while hypervirulent CG23 emerged more than 100 years earlier, in the late 1800s [28]. Notably, the hypervirulent clone isolates were also collected over a longer time period (**S1 Fig**).

We next calculated r/m (the ratio of single nucleotide variants introduced by homologous recombination relative to those introduced by substitution mutations), which ranged from 0.02–25.50 (**Fig 2B**, **S1 Table**). With the exception of CG25, the hypervirulent clones generally exhibited lower r/m values (median 1.15), while the MDR clones trended towards higher values (as may be expected from comparison of the pairwise divergence distributions, median 5.47), although the differences were not statistically significant (Kruskall-Wallis test p = 0.07).

**Fig 3:**
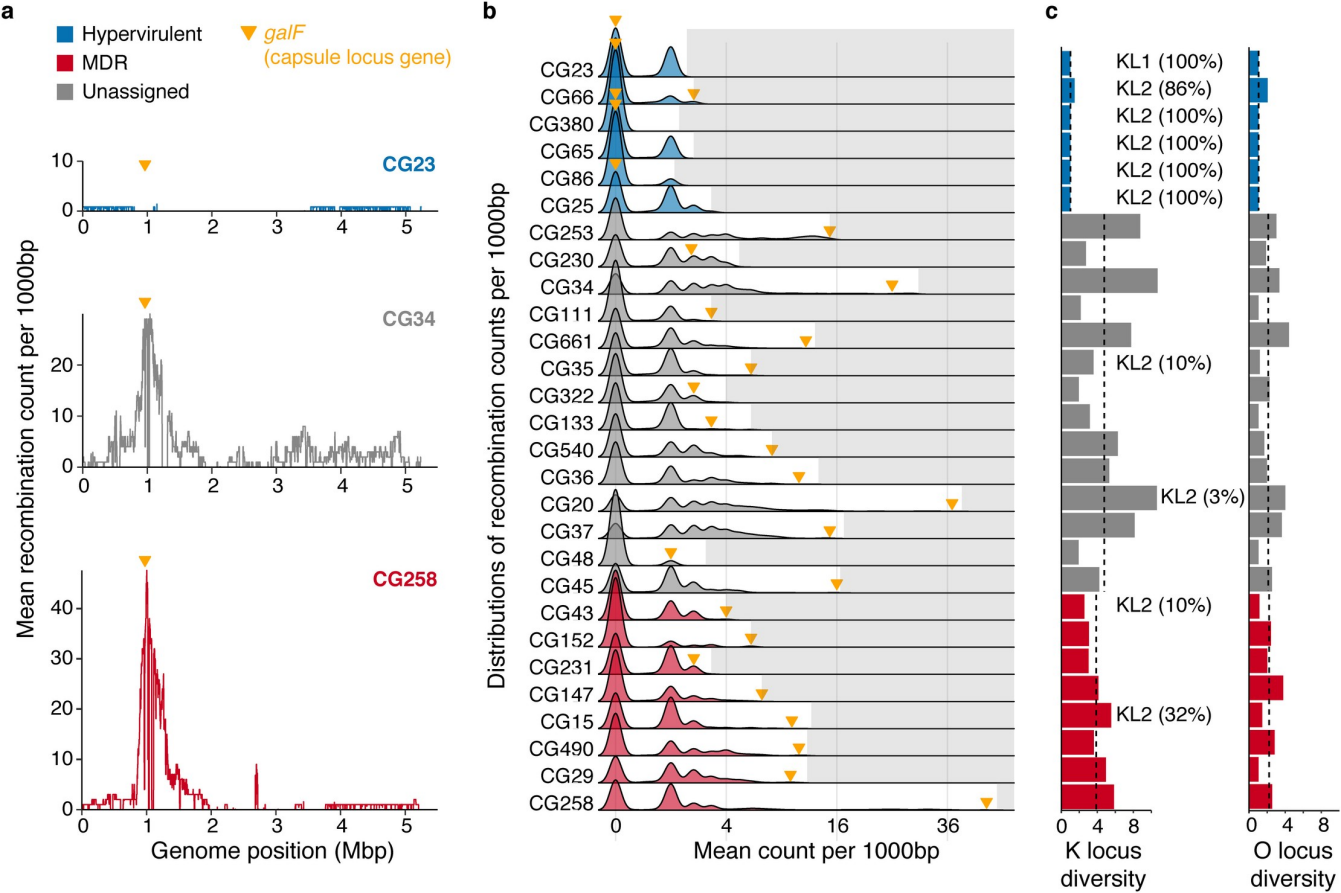
Recombination hotspots, capsule (K) and LPS antigen (O) locus diversity. **a)** Example plots showing mean recombination counts per base calculated over non-overlapping 1000 bp windows of the chromosome for hypervirulent CG23, unassigned CG34 and multi-drug resistant CG258. The latter two have a distinct peak in recombination counts around the K/O loci (marked by the yellow arrows). **b)** Density plots showing the distributions of mean recombination counts per base calculated as in **3a**. For each row, grey shading marks values outside the distribution of that clone and the yellow arrow indicates the value for the window containing *galF*, the 5’-most K locus gene. Plots are coloured by clone type as above. **c)** K and O locus diversities by clone (effective Shannon’s diversities). Clones harbouring KL1 or KL2 encoding the highly serum resistant capsule types K1 and K2, respectively are marked (numbers in parentheses indicate the percentage of successfully typed genomes harbouring the locus). Bars are coloured by clone type as above. Dashed lines indicate median values for each clone type.

Recombination events were not evenly distributed across chromosomes: in 19/28 clones ≥50% of the chromosome was not subject to any recombination events, while the maximum recombination load in each clone ranged from mean 1.1–47.6 events (**Fig 3A and 3B**, **S4 Fig**). In many cases there was a major peak defining a recombination hot-spot at the capsule (K) and adjacent LPS antigen (O) biosynthesis loci (see e.g. CG34 and CG258 in **Fig 3A**, and **S4 Fig**). Among the 17 clones with ≥1 detectable recombination hotspot (arbitrarily defined as mean recombination count of ≥5 per base calculated over non-overlapping 1000 bp windows), the *galF* K locus gene was ranked among the top 2% recombination counts in 16 clones (**Fig 3B** and **S4 Fig**). Consistent with these findings, 20 clones were associated with ≥3 distinct K loci and 11 clones were also associated with ≥3 O loci (**Fig 3C**, **S5 and S6 Figs**). The absolute numbers of K and O-loci per clone were significantly associated with sample size (p < 1x10^-4^ and p = 0.0157, respectively), but this was not the case for the respective diversity metrics shown in **Fig 3C** (effective Shannon diversity, p = 0.259 and p = 0.56). Therefore the data indicate that the capsule and LPS are subject to strong diversifying selection and that the greater the number of samples, the greater the likelihood of capturing additional K or O loci. However, this was not the case for the hypervirulent clones, which were associated with low K and O locus diversity: five out of the six had just one K and one O locus type (either KL1 or KL2, plus O1/O2v1 or O1/O2v2) and showed no evidence of recombination events affecting *galF* (**Fig 3B and 3C, S5 and S6 Figs**).

The key selective drivers for K/O locus diversity are not known. The role of the mammalian immune system is unclear because *K*. *pneumoniae* are opportunistic rather than obligate human pathogens that can inhabit a range of host-associated niches and live ubiquitously in the environment [37]. Mammalian gastrointestinal carriage of *K*. *pneumoniae* is common [38], and may provide some immune exposure favouring capsule diversity. However, phage and/or protist predation likely also play a role [39]. Numerous capsule specific *K*. *pneumoniae* phage have been reported [40,41] and ecological modelling supports a key role for phage-induced selective pressures in maintaining surface polysaccharide diversity in free-living bacteria [42].

In either scenario, the relative lack of capsule diversity among the hypervirulent clones may suggest that they are not subject to the same selective pressures, perhaps indicating some sort of ecological segregation e.g. preference for host- vs non-host associated environments, for different hosts or differences in the duration of host carriage. In particular, given the incidence of disease we might predict that hypervirulent clones are carried in the human gastrointestinal tract less frequently and/or for shorter duration than MDR clones. This possibility is intriguing and could explain the separation of hypervirulence and MDR, by limiting opportunities for horizontal gene transfer between MDR and hypervirulent clones. Isolates representing both clone types have been identified among diverse host-associated niches including the human gastrointestinal tract [28,38,43] but it is not possible to determine any particular ecological preference or differences in carriage duration due to the lack of systematic sampling efforts to-date. An alternative explanation is that the hypervirulent clones are subject to some sort of mechanistic limitation for chromosomal recombination, that in turn limits surface polysaccharide diversity and the acquisition of other chromosomally encoded accessory genes, as have recently shown to be frequently acquired by CG258 strains [44]. If so, we may also expect a general trend towards lower gene content diversity in the hypervirulent clones.

### Hypervirulent clones are associated with low pan-genome diversity

**Fig 4:**
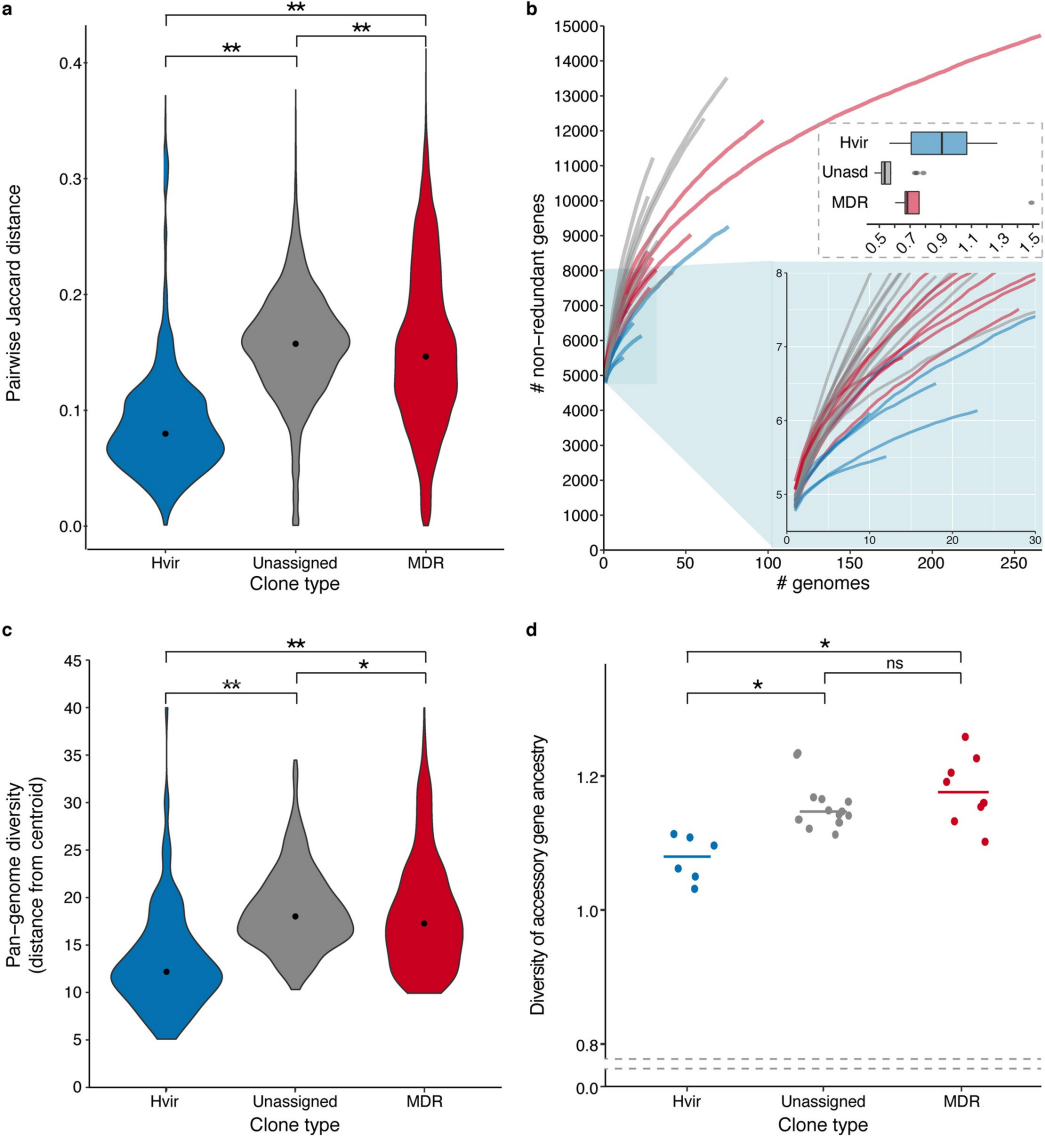
Gene content diversity. **a)** Pairwise gene content Jaccard distances were calculated for all pairs of genomes within each clone and are summarised by clone type (n = 7150, 15754, 86228 pairs for the hypervirulent, unassigned and MDR groups, respectively). Black points indicate median values. **b)** Gene accumulation curves were generated independently for each clone using the rarefy function in the R Vegan [91] package to analyse each gene content matrix, and are coloured by clone type. The upper inset box shows the distributions of alpha values. The lower inset box shows a magnified view for up to 30 genomes. **c)** Violin plots showing the distributions of Euclidean distances from clone centroids for each genome, calculated from the gene content matrix after decomposition to 463 dimensions (n = 155, 390 and 547 for the hypervirulent, unassigned and MDR groups, respectively). Black points indicate median values. **d)** Scatter plot showing ancestral diversity of accessory genes for each clone grouped by clone type. Accessory genes were identified as those present in <95% genomes. Each gene was assigned to a putative ancestral origin using Kraken v0.10.6, genus level assignments were used to calculate Shannon’s diversity indices (n = 6, 14 and 8 for the hypervirulent, unassigned and MDR groups, respectively). Horizontal lines indicate median values. Note that the y-axis is broken. For all panels, brackets indicate Wilcoxon Rank Sum tests of pairwise group comparisons; ns, not significant; *, p < 0.05; **, p < 1x10^-15^.

To assess overall gene content diversity we conducted a pan-genome analysis using Roary [45]. Given the evidence that our hypervirulent clones represented a greater amount of evolutionary divergence than the MDR clones (**Fig 2A**), we might expect these clones to harbour comparatively greater gene content diversity. Contrary to this expectation, pairwise Jaccard gene content distances were generally lower for genome pairs within hypervirulent clones than the MDR or unassigned clones, suggesting the former have less diverse pan-genomes than expected (p < 1x10^-15^ for each pairwise Wilcoxon Rank Sum test, **Fig 4A**, **S7A Fig**). Supporting this trend, the hypervirulent clones were associated with comparatively shallow pan-genome accumulation curves (**Fig 4B).** In order to quantify the differences in these curves we fitted the pan-genome model proposed by Tettelin and colleagues [46], and derived an alpha value for each clone (**S1 Table**), whereby values <1 indicate an open pan-genome and >1 indicate a closed pan-genome. Consistent with previous data showing extensive gene content diversity within the *K*. *pneumoniae* species [1], the majority of clones had alpha values below 1. The exceptions were hypervirulent clones CG380, CG66 and CG25 plus MDR clone CG231, however we note that the standard error of these estimates ranged from 0.25 to 0.65. There was a trend towards higher alpha values (i.e. less open) among the hypervirulent clones (median alpha = 0.91, IQR 0.71–1.07), in comparison to the unassigned clones (median alpha = 0.53, IQR 0.51–0.58, p = 0.0033) and the MDR clones (median alpha = 0.68, IQR 0.67–0.76, p = 0.345) (**Fig 4B**), but only the former was significant.

It is well known that large groups of accessory genes can be linked on the same mobile element (e.g. large conjugative MDR plasmids that are common in the MDR clones, or the virulence plasmids characteristic of the hypervirulent clones), so a single gain or loss event may have a large effect on gene-based measures such as pairwise Jaccard distances and accumulation curves. Hence we used a principal component analysis (PCA) to generate a metric that is less sensitive to the correlation structure in the gene content data (see **Methods**). The PCA transformed the accessory gene content matrix comprising 1092 genomes vs 39375 genes into coordinates in a 463-dimensional space. These 463 axes accounted for 95% of the variation in the data and were used to calculate the Euclidean distance of each genome to its clone centroid. The resulting distributions of distances provided further support that the MDR and unassigned clones display greater gene content variation than hypervirulent clones (**Fig 4C;** p < 1x10^-15^, 2 d.f., Kruskal-Wallis test; p < 1x10^-15^ for each pairwise Wilcoxon Rank Sum test) and suggest this is associated with a greater frequency of horizontal gene transfer events rather than a similar number of events introducing larger changes in gene content. In addition, the putative ancestry of accessory genes (see **Methods**) was more diverse among MDR and unassigned clones than the hypervirulent clones, supporting that the latter are subject to a more limited range of partners for horizontal gene transfer (Wilcoxon Rank Sum tests: hypervirulent vs MDR, p = 0.0027; hypervirulent vs unassigned, p = 1x10^-4^; MDR vs unassigned, p = 0.38; **Fig 4D, S1 Table**).

The data suggest that the unassigned clones may harbour slightly greater gene content diversity than the MDR clones (median pairwise Jaccard distance 0.1574 vs 0.1464, p < 1x10^-15^; median Euclidean distance 18.01 vs 17.26, p = 0.0083; alpha values 0.53 vs 0.68, p = 0.0064), which may indicate that the former inhabit a more generalist niche than the MDR clones e.g. inhabit a greater variety of hosts, or that the MDR clone samples represent more recent clonal expansions. The latter is consistent with the lower nucleotide divergence captured in the MDR compared to the unassigned clones (**Fig 2A**), although we note that our analysis did not support nucleotide divergence as a major driver of differences in gene content variation (**S1 Text and S3 Table**).

### Plasmid and phage diversity differs between clones

**Fig 5:**
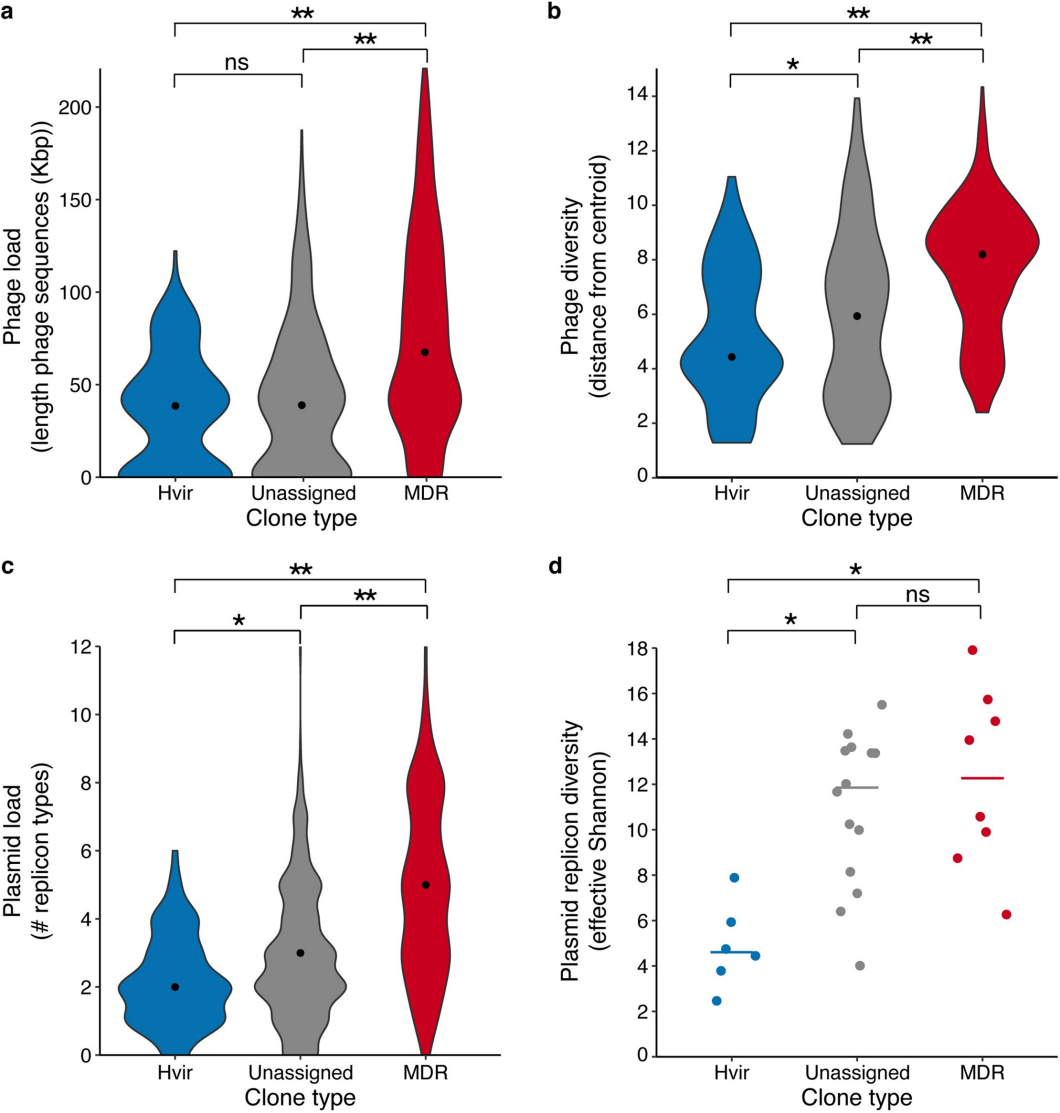
Phage and plasmid diversity. **a)** Violin plots showing the distributions of the total length (kbp) of phage sequence identified per genome. **b)** Violin plots showing the distributions of Euclidean distance to clone centroids calculated from the phage gene content matrix decomposed into 210 dimensions. **c)** Violin plots showing the distributions of plasmid replicon count per genome (note that perfectly co-occurring replicons are counted once only). **d)** Effective Shannon’s diversities for plasmid replicons, by clone. Horizontal lines indicate median values (n = 6, 14 and 8 for the hypervirulent, unassigned and MDR groups, respectively). For all violin plots, data points represent individual genomes (n = 155, 390 and 547 for the hypervirulent, unassigned and MDR groups, respectively) and black points indicate median values. For all panels, brackets indicate Wilcoxon Rank Sum tests of pairwise group comparisons; ns, not significant; *, p < 0.01; **, p < 1x10^-15^.

To further explore differences in common sources of accessory gene diversity, we assessed phage and plasmid diversity. For each genome we summed the length of genomic regions identified as phage by VirSorter [47] (range 0–221 kbp, **S2 Table**) and used a PCA of phage-associated gene content to calculate distance to clone centroids as for the total pan-genome (**Fig 5A and 5B** and **S8 Fig**). Hypervirulent clones showed similar phage load and slightly reduced diversity compared to the unassigned clones (**Fig 5A and 5B**; Wilcoxon Rank Sum tests, hypervirulent vs unassigned: load, p = 0.15; diversity, p = 0.0165). MDR clones were associated with higher load and diversity than both the hypervirulent and unassigned groups (p < 1x10^-15^ for each pairwise comparison). Although these analyses were dependent on the quality and breadth of the underlying viral sequence database which may be subject to species bias, we have no reason to expect that this would be skewed with respect to MDR over hypervirulent clones. Hence it is clear that *K*. *pneumoniae*, and in particular the MDR clones, are subject to frequent attack by diverse phage.

Unfortunately it is not possible to reliably identify plasmid sequences from draft genome assemblies [48]. Instead we used plasmid replicon and relaxase (*mob*) typing as indicators of plasmid load and diversity. Each genome contained 0–12 of 69 uniquely distributed replicon markers and 0–23 *mob*-positive assembly contigs (detected by screening against the PlasmidFinder database [49] and *mob* PSI-BLAST [50,51], respectively; **S2 Table**). MDR and unassigned genomes harboured a greater number of replicon markers than hypervirulent genomes, largely driven by low replicon loads in CG23 that was overrepresented among the hypervirulent genomes (**Fig 5C and 5D and S9 Fig**; Wilcoxon Rank Sum tests: hypervirulent vs unassigned, p = 1.5×10^−4^; MDR vs unassigned, p < 1x10^-15^; MDR vs hypervirulent, p < 1x10^-15^). There were no significant differences between the hypervirulent and unassigned groups for counts of *mob-*positive contigs per genome, and comparatively small differences between the MDR and unassigned or hypervirulent groups (Wilcoxon Rank Sum test: MDR vs unassigned, p = 1x10^-6^; MDR vs hypervirulent, p <1x10^-6^, **S9 Fig**).

Comparison of effective Shannon’s diversity of replicon profiles indicated that the hypervirulent clones harbour less plasmid diversity than either of the unassigned or MDR clones (not driven solely by CG23, see **Fig 5D, S9 Fig,** Wilcoxon Rank Sum tests: hypervirulent vs MDR, p = 0.0013; hypervirulent vs unassigned, p = 0.0015; MDR vs unassigned, p = 0.44). Note that sample size was also significantly associated with plasmid replicon diversity (p = 0.017) but that both variables remained significant in a combined model (p<0.023, see **S1 Text and S3 Table**). Similar trends were seen for effective Shannon’s diversity of *mob* types but the differences were not statistically significant after Bonferroni correction for multiple testing (n = 3 tests), a finding that is not surprising given that far fewer *mob* types have been defined and that only ~48% completely sequenced Enterobacteriaceae plasmids deposited in GenBank could be *mob* typed (whereas ~83% could be replicon typed) [50].

While these data are subject to the biases of the underlying databases within which clinically relevant (MDR and virulence) plasmids are overrepresented, they are also consistent with the findings above regarding overall pan-genome diversity. These data imply that MDR and unassigned clones frequently acquire and lose plasmids, consistent with the high plasmid diversity reported previously for ST258 [36,52] and several others [4], and with data from recent investigations of *K*. *pneumoniae* circulating in hospitals which showed that individual plasmids transferred frequently between clones [53,54].

In contrast, the data showed that hypervirulent clones were associated with comparatively low plasmid diversity, mirrored by generally narrow plasmid load distributions (with CG25 as a putative exception, see **S1 Text**). Taken together these data imply that hypervirulent clones acquire novel plasmids infrequently but can stably maintain them. For example, as noted above we recently estimated that the virulence plasmid, which by definition is highly prevalent in these clones, has been maintained for >100 years in CG23 [28]. In addition, laboratory passage experiments have shown that hypervirulent strains can maintain MDR plasmids introduced *in vitro* [26], and we showed that a horse-associated subclade of CG23 has maintained a single MDR plasmid for at least 20 years [28].

The data also indicate that MDR clones may carry higher numbers of plasmids than either unassigned or hypervirulent clones. *In vitro* studies with *Escherichia coli* have shown that the relative fitness cost of a single plasmid can vary in a strain-dependent manner [55]. Hence it is possible that the higher plasmid loads detected here in MDR *K*. *pneumoniae* clones reflect a lower cost of plasmid carriage, perhaps due to compensatory mutation(s) on the chromosome e.g. in DNA helicase and RNA polymerase genes as have been shown to influence plasmid carriage in *Pseudomonas* [56].

### The polysaccharide capsule as a constraint on horizontal gene transfer

The combination of infrequent plasmid acquisitions and limited chromosomal recombination suggests that hypervirulent clones may be subject to particular constraints on DNA uptake and/or integration. One possible explanation is that these *K*. *pneumoniae* clones possess enhanced defences against incoming DNA such as CRISPR/Cas or restriction-modification (R-M) systems. However, our genome data reveals no significant differences in either system (see **S1 Text**, **S10–S13 Figs**). Alternatively, the key virulence determinants themselves, or other proteins encoded on the virulence plasmid, may play a role. Two variants of the virulence plasmid predominate among hypervirulent clones and share limited homology aside from the *iuc*, *iro* and *rmpA* loci [24]. It seems unlikely that a siderophore system would influence DNA uptake, however it is conceivable that upregulation of capsule expression by *rmpA* [15,57] may play a role by exacerbating the inhibitory effect of the capsule.

Capsule expression has been associated with a comparative reduction in *K*. *pneumoniae* transformation frequency *in vitro* [58] and in a natural *Streptococcus pneumoniae* population [59]. Additionally, the capsule is known to conceal the LPS [60] which, together with the OmpA porins, are considered key target sites for attachment of conjugative pili during the initial phases of mate-pair formation [61,62]. Hence we speculate that overexpression of the capsule in hypervirulent clones may result in a reduction of DNA uptake. Given that capsule types differ substantially in their thickness and polysaccharide composition [15,63], it is also likely that their influence on DNA uptake is type dependent. The K2 capsule, which is associated with five of the 6 hypervirulent clones investigated here is considered among the thicker capsule types [15] and thus may have a comparatively greater influence. We used our genome data to test this hypothesis by comparing the genomic diversity of KL2 and non-KL2 genomes within MDR CG15, the only clone with sufficient KL2 and non-KL2 genomes for comparison. CG15-KL2 genomes formed a deep branching monophyletic subclade consistent with long-term maintenance of KL2 for an estimated 34 years (see **S1 Text, and S14 and S15 Figs**). This KL2 subclade showed a low rate of recombination compared to the rest of the clone (r/m 0.58 vs 6.75). The distribution of pairwise Jaccard gene distances also supported lower gene content diversity among the CG15-KL2 subclade (p < 1x10^-12^), as did the pan-genome curve alpha values (CG15-KL2; 0.785 vs CG15-other; 0.686), but this was not supported by the pan-genome PCA analysis (p = 0.2451, **S1 Text and S14 Fig**). Thus the genome data provide some support for our hypothesis that should motivate future laboratory studies of this phenomenon; a task that will not be trivial given the low efficiency of *in vitro* transformation for wild-type *K*. *pneumoniae* strains [64], the challenge of identifying suitable selective markers for distinguishing MDR strains, and the sensitivity of conjugation efficiencies to laboratory growth conditions [61]. If true, this would imply that hypervirulent clones are evolutionarily constrained by a key determinant of the hypervirulent phenotype, and as such are self-limited in their ability to adapt to antimicrobial pressure.

Conversely, it is possible that the differences identified here do not reflect a mechanistic barrier for DNA uptake in hypervirulent clones, but rather ecological characteristics and/or mechanistic features common to MDR or unassigned clones (e.g. exposure to unique selective pressures due to preference for a specific ecological niche or enhanced tolerance of plasmid fitness costs, as discussed above). In particular, the former may also explain the elevated phage load and diversity in the MDR clones compared to either the hypervirulent or unassigned clones. However, as discussed above there are currently insufficient data to address these hypotheses.

### Conclusions

Regardless of the mechanisms, our data clearly show that hypervirulent *K*. *pneumoniae* clones are less diverse than their MDR counterparts, and suggest that the rate of virulence plasmid acquisition by MDR clones will far exceed the rate of MDR plasmid acquisition by hypervirulent clones. This is particularly worrying from a hospital infection control perspective since many of the MDR clones investigated here appear well adapted to transmission and colonisation in the human population, and are frequent causes of hospital outbreaks [4,5]. Given the mounting evidence that MDR clones can carry multiple plasmids at limited fitness cost [65–67] and frequently exchange plasmids with other bacteria [53,54], it seems these MDR clones may also be the perfect hosts for consolidation and onwards dissemination of MDR and virulence determinants. The greatest concern is that these determinants will be consolidated onto a single mobile genetic element; indeed mosaic *K*. *pneumoniae* plasmids carrying AMR genes plus *iuc* and *rmpA2* have already been reported in two MDR *K*. *pneumoniae* clones [27,68], and *Escherichia coli* plasmids bearing *iuc*, *rmpA* and AMR genes have been detected in *K*. *pneumoniae* [24]. Whether these convergent strains and plasmids are fit and disseminating is not known. Recent experience with convergent carbapenem-resistant CG258 in China–which retrospective surveillance studies showed was already widely disseminated at the time of the outbreak report [31,69]–highlights the ease with which deadly strains can circulate unnoticed. As reports of convergent *K*. *pneumoniae* strains continue to increase, the need for global genomic surveillance encompassing clone, AMR and virulence locus information [70] is clearly greater than ever.

## Materials and methods

### Genome collection and clone definition

We collected and curated 2265 *K*. *pneumoniae* genomes, comprising 647 genomes sequenced and published previously by our group [1,12,13,71] plus 1623 publicly available genomes [36,72–78] as described previously [13]. Genomes were assigned to chromosomal multi-locus sequence types (MLST, as below), and a single representative of each sequence type (ST, n = 509) was selected for initial phylogenetics to define clones for further analysis. Sequence reads were mapped to the NTUH-K2044 reference chromosome (accession: NC_012731) using Bowtie v2 [79] and single nucleotide variants were identified with SAMtools v1.3.1[80] as implemented in the RedDog pipeline (https://github.com/katholt/RedDog). Where genomes were available only as *de novo* assemblies, sequence reads were simulated using SAMtools wgsim [80] (n = 852 genomes, for each of which 2 million x 100bp PE reads were simulated without errors). Allele calls were filtered to exclude sites that did not meet the following quality criteria: unambiguous consensus base calls, phred quality ≥30, depth ≥5 reads but <2-fold mean read depth, no evidence of strand bias. Subsequently, we generated a variable site alignment by concatenating nucleotides at core genome positions, i.e. at positions for which ≥95% genomes contained a base-call with phred quality ≥20 as we have described previously [28,81]. The resulting alignment of 192,433 variable sites was used to infer a maximum likelihood phylogeny with FastTree v2.1.9 [82] (gamma distribution of rate heterogeneity among sites, **Fig 1A**). Genomes were clustered into 259 phylogenetic lineages (clones) using patristic distance (distance threshold = 0.04). This threshold was selected because the resulting clusters included groups of STs that have been recognised as distinct clonal groups in previous genomics studies [1,2,36] and are considered in the literature as cohesive groups that can be distinguished by their clinical behaviours e.g. causing hypervirulent vs opportunistic infections. A total of 29 clones (clonal groups, CGs) were identified that were each represented by ≥10 isolates from at least three different countries. One of these (CG82) was subsequently excluded because it uniquely included only historical isolate genomes (dated 1932–1949 or unknown). The remaining 28 clones (totalling 1092 genomes) were subjected to comparative analysis in this study. We refer to each as CGX, where X is the predominant ST in the clone, as per the convention for *K*. *pneumoniae*.

For each clone of interest, reads were mapped to a completed chromosomal reference genome belonging to that clone (see **S1 Table** and below), and variant calling and phylogenetic inference was performed as above. Phylogenies were manually inspected alongside genome source information to identify and de-duplicate clusters of closely related genomes from the same patient and/or known hospital outbreaks. Additional random sub-sampling was applied to CG258, which was otherwise drastically overrepresented in the collection (>700 genomes subsampled to 266 genomes). The final set of clones and genomes used for analyses are listed in **S1 and S2 Tables**, respectively.

Note that for the investigations of virulence and AMR determinant distributions in the broader *K*. *pneumoniae* population (shown in **S2 Fig**) we considered an independent subset of the original curated genome collection (n = 1124). This subset was described previously [13] and was considered more representative of the population diversity because known outbreaks and overrepresented sequence types were subsampled.

### Clonal reference genome selection

Reference genomes for each clone were identified among publicly available completed *K*. *pneumoniae* chromosome sequences for each ST represented in the clones of interest. Where there was no suitable publicly available reference genome we selected a representative isolate from our collection, for which Illumina data were available, and generated additional long read sequence data for completion of the genome through hybrid genome assembly (details below). The exception was CG380 for which no suitable reference was publicly available and for which we did not have access to any isolates in our collection. As such, we generated a pseudo-chromosomal reference by scaffolding the *de novo* assembly contigs for genome SRR2098675 [78] (the CG380 genome with the lowest number of contigs) to the most closely related completed genome in our initial phylogeny (NCTC9136, available via the NCTC3000 genomes project website: http://www.sanger.ac.uk/resources/downloads/bacteria/nctc/). Contigs were scaffolded using Abacas [83] and manually inspected using ACT [84] (contig coverage ≥20%).

### Long read sequencing and hybrid genome assembly

Novel completed reference genomes were generated for 10 clones (CG25, CG29, CG36, CG253, CG43, CG45, CG152, CG230, CG231, CG661) for which Illumina data were available [1,85]. Novel long read data were generated on the Pacific Biosciences platform for two isolates (CG231 strain MSB1_8A, and CG29 strain INF206), and an Oxford Nanopore Technologies MinION device for the remaining isolates as described previously [86]. Long read sequence data were combined with the existing short read Illumina data to generate complete hybrid assemblies with Unicycler [87]. The final completed assemblies were deposited in GenBank (accessions listed in **S1 Table)** and are available in Figshare (see below).

### MLST, virulence and resistance gene screening

Chromosomal MLST, AMR and virulence genes were detected with SRST2 [88] (or Kleborate, available at https://github.com/katholt/Kleborate, for typing assemblies when no sequence reads were available). Sequence reads were assembled *de novo* using SPAdes v3 [89], and Kaptive v0.5.1 [13,90] was used to determine K and O locus types from assemblies. K and O locus diversities were calculated using the R package Vegan v2.4.3 [91]. The indices were converted to effective values to enable direct comparison between clones using the formula described previously [92]; effective Shannon diversity = exp(Shannon diversity).

### Recombination detection

Recombination analysis was performed independently for each clone: single nucleotide variants were identified by mapping and variant calling against the clonal reference genome as above, and a pseudo-chromosomal alignment was used as input for Gubbins v2.0.0 [35], with the weighted Robinson-Foulds convergence method and RAxML [93] phylogeny inference. The Gubbins output files were used to calculate r/m and mean recombination counts per base, calculated over non-overlapping 1000 bp windows (relative to each clone-specific reference chromosome). Pairwise nucleotide divergence across clone-specific core genome regions was calculated for each pair of genomes within a clone before and after removal of putative recombinant regions.

### Pan-genome, plasmid and phage analyses

The SPAdes derived genome assemblies were annotated with Prokka v1.11 [94] and subjected to a pan-genome analysis with Roary v3.6.0 [45] (BLASTp identity ≥95%, no splitting of ‘paralogs’). The resulting gene content matrix comprised 1092 genomes vs 39375 genes (after excluding 1070 core genes present in ≥95% genomes) was used to calculate pairwise Jaccard distances and as input for PCA with the Adegenet R package v2.0.1 [95]. Coordinates for 463 principal components (PC) were extracted, accounting for 95% of the variation in the sample. We calculated the Euclidean distance from each genome to its clone centroid (the vector of mean coordinate values for that clone, see **S16 Fig**), and compared the distributions of distances across clones. Pan-genome accumulation curves were visualised using the R package Vegan v2.4.3 [91] and alpha values were calculated by fitting the linear model log(y) ~ β*log(x) where y is the number of unique genes accumulated upon addition of a new genome (median from 100 random permutations) and x is the number of genomes. Alpha is estimated as -β, as derived from Heap’s law.

Accessory genes were identified as those present in <95% of all 1092 genomes belonging to the 28 clones analysed as described previously [1]. For each genome assembly within a clone, the accessory gene sequences were extracted and concatenated into a single multi-fasta file (one per clone) that was used as input for ancestral assignment by Kraken v0.10.6, run with the miniKraken database [96]. For each clone the proportions of accessory genes assigned to distinct genera were used to calculate Shannon’s diversity indices using the R package Vegan v2.4.3 [91].

Phage were identified from genome assemblies using VirSorter v1.0.3 [47], with the highest confidence threshold. The resulting output includes a set of putative phage sequences in fasta format, in which we identified open reading frames (ORFs) using Prokka v1.11 [94]. The resulting ORF sequences were clustered into non-redundant phage gene sequences using CD-HIT-EST v4.6.1 [97] (identity ≥95%). BLASTn was used to tabulate the presence/absence of each of the resulting phage genes within the putative phage sequences identified by VirSorter in each genome (identity ≥95%, coverage ≥95%). The resulting gene content matrix was used as input for PCA and centroid distance calculations as described above for all accessory genes.

Plasmid replicons defined in the PlasmidFinder database [49] were identified from assemblies using BLASTn as per the authors’ guidelines. Identically distributed replicons were collapsed into a single entry to minimise the influence of multi-replicon plasmids. Plasmid *mob* types were identified by PSI-BLAST as previously described [50,51]. Effective Shannon’s diversities were calculated for each clone based on the replicon and *mob* presence/absence matrices, using the R package Vegan v2.4.3 [91] as described above.

### CRISPR/Cas and restriction-modification systems

CRISPR arrays were identified from genome assemblies using the CRISPR Recognition Tool v1.2 [98]. The majority of genomes harboured no more than two arrays and hence those identified with >3 putative arrays were investigated manually to check for spurious identifications and/or identifications of single arrays split over multiple assembly contigs. Nucleotide sequences for the previously described *K*. *pneumoniae cas* genes [99] were extracted from the NTUH-K2044 reference chromosome (accession: NC_012731.1). Genomes were screened for novel *cas* genes by HMM domain search using the domain profiles developed by Burstein and colleagues [100] (HMMER v3.1b2, bit score ≥200 [101]). A representative set of putative novel *cas* genes was extracted from the genome of isolate INF256 (read accession: ERR1008719, genome assembly available in Figshare) and tBLASTx was used to detect the presence of NTUH-K2044- and INF256-like *cas* genes among all genomes (identity ≥85%, coverage ≥25%). Note that the INF256 *cas* genes were subsequently found to be highly similar to those of strain Kp52.145 reported during the course of this study [102].

Putative restriction enzymes (REases) were identified from genome assemblies by HMM domain search using the domain profiles developed previously [103,104], parameters as above. CD-HIT v4.6.1 [97] was used to cluster the predicted amino acid sequences of these REases such that distinct clusters represented enzymes that are thought to recognise distinct methylated nucleotide motifs [103]; i.e. using amino acid identity thresholds of 80% identity for type I and type III REases; 55% identity for type II REases. To date no suitable threshold has been determined for type IV REases and thus in order to include type IV enzymes in our analyses we used the more conservative 55% identity threshold. Type IIC REase sequences cannot be aligned [103] and were therefore excluded from the analysis. Nucleotide sequences for a single representative of each REase cluster were used to search all assemblies by BLASTn (identity ≥80%, coverage ≥90%). Only the single best hit was recorded for each region of each genome. In order to assess the DNA recipient potential of each genome, we also used BLASTn to screen a broader sample of the *K*. *pneumoniae* population, comprising the 1124 representative non-redundant genomes from our curated collection as described above and previously [13], plus a further 598 diverse *K*. *pneumoniae* genomes published during the course of this project [105–108]. A putative donor-recipient pairing was considered compatible if the complete set of REases in the recipient genome were also present in the donor genome (we assume that genomes positive for an REase also carry the corresponding methyltransferase).

### Investigating the impact of the K2 capsule on CG15 recombination and pan-genome diversity

K loci were overlaid onto the CG15 recombination-free maximum likelihood phylogeny, revealing that the KL2 locus was restricted to one of two major subclades (shaded blue and grey in **S14 Fig**). Recombination dynamics and pan-genome diversity were investigated separately for each of these subclades, using the methods described above. We used BEAST2 [109] to estimate the time to most recent common ancestor (tMRCA) of the KL2 subclade, using as input the recombination-free single nucleotide variant alignment generated by Gubbins (2967 bp). The final analysis included 21 genomes (those for which years of collection were not known were excluded, see **S2 Table**) and was completed as described previously [28]. Temporal structure was confirmed by date-randomisation tests, which showed that the evolutionary rate derived from the true data did not overlap those derived from any of 20 independent randomisations (**S15 Fig**).

## Supporting information

S1 Table Summarised sample information and genomic analysis results for the 28 *K*. *pneumoniae* clones investigated in this study. (XLSX)

S2 Table Sample information and genotyping results for the 1092 *K*. *pneumoniae* genomes investigated in this study. (XLSX)

S3 Table Outcomes of statistical tests to assess the influence of sampling bias. (PDF)

S1 Text Supplementary results details. (PDF)

S1 Fig Sample years and geographic continents of collection. **a)** Density plots showing the distribution of years of collection for all isolates for which years were known (see **S1 and S2 Tables**). Distributions are coloured by clone type; blue, hypervirulent; grey, unassigned; red, multi-drug resistant. **b)** Effective Shannon’s diversity of continent of collection. Bars are coloured by clone type as in **(a)**. **c)** Count of isolates represented in each clone, coloured by continent of collection as indicated.
(TIF)

S2 Fig Number of acquired resistance genes per genome. Density plots and matched boxplots show data for 1124 genomes that can be considered representative of the broader population of genomes (i.e. with isolates from known outbreaks and overrepresented clones subsampled, as described previously in Wyres *et al MGen* 2016). Data are stratified by virulence status, defined as follows: No ICE*Kp* or VP = none of the yersiniabactin, colibactin or aerobactin loci were identified, n = 781 (the former two are indicative of the ICE*Kp* and the latter is indicative of the virulence plasmid, VP); ICE*Kp* only *=* the yersiniabactin synthesis locus plus/minus the colibactin synthesis locus was identified without the aerobactin locus, n = 255; VP +/- ICE*Kp* = the aerobactin locus was identified plus/minus the yersiniabactin and/or colibactin loci, n = 88.
(TIF)

S3 Fig **Distributions of pairwise nucleotide divergence before (a) and after (b) removal of recombinant sequence regions.** Boxplots are coloured by clone type; blue, hypervirulent; grey, unassigned; red, multi-drug resistant.
(TIF)

S4 Fig Chromosomal distribution of recombination events in 28 distinct clones. Mean recombination events per base (calculated across non-overlapping 1000 bp windows, relative to the clone reference chromosome—see **S1 Table**) are shown for each clone, ordered from left-to-right by row as in **Fig 1B and 1C**. Y-axes represent mean counts (note that scales differ), and x-axes represent genome position relative to the reference genome for the given clone (coordinates in Mbp). Chromosomes are aligned such that the *galF* K locus gene starts at 1 Mbp. Grey dashed lines indicate mean = 5 recombination events. Clone labels are coloured by clone type; blue, hypervirulent; grey, unassigned; red, multi-drug resistant.
(TIF)

S5 Fig Distribution of K loci across clones. K locus assignments for which the Kaptive match confidence was “Good” or better are shown. Low or no confidence matches were grouped as “Unknown.” Data points are scaled proportional to the number of genomes they represent and coloured by clone type as indicated.
(TIF)

S6 Fig Distribution of O loci across clones. O locus assignments for which the Kaptive match confidence was “Good” or better are shown. Low or no confidence matches were grouped as “Unknown.” Data points are scaled proportional to the number of genomes they represent and coloured by clone type as indicated. The O1 and O2 lipopolysaccharides are both associated with two distinct O locus variants (v1 and v2). Distinction between the O1 and O2 phenotypes is dependent on the presence of genes located elsewhere in the genome (not shown).
(TIF)

S7 Fig Pan-genome diversity by clone. **a)** Jaccard gene distances. Data points within each distribution represent pairwise comparisons for each pair of genomes within the clone. **b)** Euclidean distances from clone centroids calculated from the pan-genome gene content matrix after decomposition to 463 dimensions (see **Methods**). Data points within each distribution represent single genomes. In both panels boxplots are coloured by clone type; blue, hypervirulent; grey, unassigned; red, multi-drug resistant.
(TIF)

S8 Fig Phage loads and gene content diversity by clone. **a)** Boxplots show the distributions of the total length (kbp) of phage sequence identified per genome. **b)** Boxplots show the distributions of Euclidean distance to clone centroids calculated from the phage gene presence matrix decomposed into 210 dimensions.
(TIF)

S9 Fig Estimated plasmid loads and diversity. **a)** Distributions of number of *mob*-positive contigs by clone type. Each data point represents a single genome, grouped by clone type. **b)** Effective Shannon’s diversity of *mob* types. Each data point represents a single clone grouped by clone type. **c)** Counts of unique plasmid replicon types (perfectly co-occurring types counted once only). **d)** Counts of *mob*-positive genome assembly contigs. Each data point in **(c)** and **(d)** represents a single genome. Clones are coloured by clone type as indicated. For panels **(a)** and **(b)**, brackets indicate Wilcoxon Rank Sum tests of pairwise group comparisons; ns, not significant; *, p < 0.01.
(TIF)

S10 Fig Distribution of CRISPR/Cas systems across clones. **a)** Proportion of genomes harbouring putative intact CRISPR/Cas loci i.e. those harbouring at least one CRISPR array and a complete set of 8 *cas* genes. Bars are coloured by clone type; blue, hypervirulent; grey, unassigned; red, multi-drug resistant. **b)** Proportion of genomes harbouring 1, 2, or 3 CRISPR arrays (upper) and proportion of genomes harbouring at least one *cas* gene of types A (NTUH-K2044-like) or B (Kp52.145-like). White, 0 genomes; dark grey, all genomes.
(TIF)

S11 Fig Distribution of REase counts per genome. Violin plots show the distributions of the number of distinct REases identified in each genome; **a)** Type I REases; **b)** Type II REases; **c)** Type III REases; **d)** Type IV REases. Brackets indicate Wilcoxon Rank Sum tests for pairwise comparisons; ns, not significant after Bonferroni multiple testing correction; ▭, p < 0.0042; *, p < 0.001; **, p < 1x10^-15^.
(TIF)

S12 Fig Prevalence of REase genes among *K*. *pneumoniae* clones. Heatmap showing the proportion of genomes for each clone that were positive for ≥1 copy of each of 33 type I, 13 type II, 7 type III and 13 type IV REase genes (shown in rows). Clone names are coloured by clone type; blue = hypervirulent; grey = unassigned; red = multi-drug resistant.
(TIF)

S13 Fig Distributions of R-M donor-recipient compatibility by clone. Data points represent individual genomes and show the number of compatible donors from a collection of 1722 diverse *K*. *pneumoniae*.
(TIF)

S14 Fig Influence of the K2 capsule on recombination and pan-genome diversity of multi-drug resistant CG15. **a)** Recombination-free maximum-likelihood phylogeny (mid-point rooted) with tips coloured by capsule (K) locus as indicated. The CG15-KL2 (blue) and CG15-other (grey, diverse K loci) subclades are marked. The divergence date for the CG15-KL2 subclade, estimated by BEAST2 analysis of the 21 CG15-KL2 genomes with known isolation dates, was estimated to be 1970 (95% HPD, 1974–1980). **b)** Recombination events within the CG15-KL2 and CG15-other subclades. Mean recombination events per base calculated over non-overlapping 1000 bp windows of the chromosome are plotted by position in the CG15 reference genome PMK1 (accession: CP008929) adjusted such that the capsule locus is shown at 1 Mbp. **c)** and **d)** Pan-genome diversity of the CG15-KL2 and CG15-other subclades compared to the hypervirulent clones and the remaining MDR clones. **c)** Violin plots show the distribution of pairwise Jaccard gene distances. **d)** Violin plots show the distributions of Euclidean distances from clone or subclade centroids, calculated from pan-genome gene content matrix after decomposition to 463 dimensions as in **Fig 4C**. Brackets indicate Wilcoxon Rank Sum tests for pairwise comparisons; ns, not significant; *, p < 1x10^-6^; **, p < 1x10^-15^.
(TIF)

S15 Fig BEAST2 evolutionary rate estimates for CG15-KL2. The median estimates and ranges are shown for the true CG15-KL2 data (red) and CG15-KL2 sequence data with 20 independent date randomisations (grey).
(TIF)

S16 Fig Schematic representation of pan-genome euclidean distance calculations. Genomes representing one hypervirulent (blue, n = 4) and one MDR (red, n = 6) clone are shown projected in a 2D space where the position of each genome (x, y) is determined by the relevant values for principal components x and y. Clone centroids are calculated and plotted as the vector of mean coordinates i.e. (μ_x_, μ_y_). For a given genome at position (x_1_, y_1_) the distance to clone centroid can be calculated as the Euclidean distance, c: c = √(a^2^ + b^2^) = √((μ_x_−x_1_)^2^ + (μ_y_−y_1_)^2^)
(TIF)
