# Supplementary material for: Distinct evolutionary dynamics of horizontal gene transfer in drug resistant and virulent clones of Klebsiella pneumoniae
Source: PLoS Genet. 2019 Apr 15;15(4):e1008114. doi: 10.1371/journal.pgen.1008114 (PMC6483277; doi:10.1371/journal.pgen.1008114)
Supplement: S3 Table — (PDF) [file pgen.1008114.s003.pdf]

S3 Table for: Distinct evolutionary dynamics of horizontal gene transfer in drug resistant and virulent clones of *Klebsiella pneumoniae*

Kelly L Wyres[1*], Ryan R Wick[1], Louise M Judd[1], Roni Froumine[1], Alex Tokolyi[2], Claire L Gorrie[3], Margaret M C Lam[1], Sebastián Duchêne[2], Adam Jenney[4] and Kathryn E Holt[1,2,5]

[1]Department of Infectious Diseases, Monash University, Melbourne, Victoria, Australia
[2]Department of Biochemistry and Molecular Biology, Bio21 Molecular Science and Biotechnology Institute, University of Melbourne, Parkville, Victoria, Australia
[3]Department of Infectious Diseases and Microbiology Unit, The Alfred Hospital, Melbourne, Victoria, Australia
[4]Microbiological Diagnostic Unit Public Health Laboratory, Department of Microbiology and Immunology, University of Melbourne, Peter Doherty Institute for Infection and Immunity, Melbourne, Victoria, Australia
[5]London School of Hygiene and Tropical Medicine, Keppel Street, London, UK

* Corresponding author; kelly.wyres@monash.edu

S3 Table: Outcomes of statistical tests to assess the influence of sampling bias

Outcome variable	Explanatory variable		
	**Sample size**	**Geographic diversity**	**Nucleotide diversity**
	P (uncorrected) * significant after correction	P (uncorrected) * significant after correction	P (uncorrected) * significant after correction
Pairwise nucleotide diversity (before recombination removal, median)	0.235	0.054	n/a
Pairwise nucleotide diversity (after recombination removal, median)	0.282	0.101	n/a
r/m	0.742	0.295	0.200
# K-loci	**7.12×10^{-05} ***	0.392	0.291
K locus diversity	0.259	0.826	0.411
# O loci	**0.016 ***	0.781	0.541
O-locus diversity	0.560	0.612	0.962
Gene content Jaccard distance (median)	0.836	0.361	0.770
Pan-genome Euclidean distance (median)	0.448	0.996	0.519
Pan-genome curve alpha	0.429	0.320	0.299
Accessory gene ancestral diversity	0.706	0.544	0.424
Phage Euclidean distance (median)	0.023	0.253	0.5
Plasmid replicon diversity	**0.017 ***	0.022	0.173
Plasmid *mob* diversity	0.187	0.926	0.533

Variables are clone-specific summary metrics tested in independent general linear models. Explanatory variables: Sample size = total number of genomes in the clone sample; Geographic diversity = effective Shannon's diversity of continent of isolate collection (as defined in S1 Table, excluding isolates from unknown geographies); Nucleotide diversity = median pairwise nucleotide diversity after removal of recombinant regions detected by Gubbins. Uncorrected p-values are shown. * indicates significance after Bonferroni multiple testing correction (within rows, P(type 1 error) <0.05).
