# Supplementary material for: Distinct evolutionary dynamics of horizontal gene transfer in drug resistant and virulent clones of Klebsiella pneumoniae
Source: PLoS Genet. 2019 Apr 15;15(4):e1008114. doi: 10.1371/journal.pgen.1008114 (PMC6483277; doi:10.1371/journal.pgen.1008114)
Supplement: S1 Text — (PDF) [file pgen.1008114.s004.pdf]

S1 Text for: Distinct evolutionary dynamics of horizontal gene transfer in drug resistant and virulent clones of *Klebsiella pneumoniae*

Kelly L Wyres[1*], Ryan R Wick[1], Louise M Judd[1], Roni Froumine[1], Alex Tokolyi[2], Claire L Gorrie[3], Margaret M C Lam[1], Sebastián Duchêne[2], Adam Jenney[4] and Kathryn E Holt[1,2,5]

[1]Department of Infectious Diseases, Monash University, Melbourne, Victoria, Australia
[2]Department of Biochemistry and Molecular Biology, Bio21 Molecular Science and Biotechnology Institute, University of Melbourne, Parkville, Victoria, Australia
[3]Department of Infectious Diseases and Microbiology Unit, The Alfred Hospital, Melbourne, Victoria, Australia
[4]Microbiological Diagnostic Unit Public Health Laboratory, Department of Microbiology and Immunology, University of Melbourne, Peter Doherty Institute for Infection and Immunity, Melbourne, Victoria, Australia
[5]London School of Hygiene and Tropical Medicine, Keppel Street, London, UK

* Corresponding author; kelly.wyres@monash.edu

Statistical tests of the influence of clone sample size, geographic diversity and nucleotide divergence
The genome data investigated in this study represent a convenience sample derived from our genome collections augmented with publicly available data (see **Methods** and **S2 Table**). While every effort was made to limit the influence of sample bias (e.g. subsampling data from known outbreaks and exclusion of clones comprising isolates collected from fewer than three geographic regions etc), we must carefully consider the potential impact of any remaining biases. Of particular concern were the influence of differences in clone sample size, geographic diversity of isolates and the level of ancestral nucleotide divergence captured within the samples. We used a general linear model (as implemented in R v3.3.3) to test for the association between each of these explanatory variables and each of 14 clone-specific metrics (outcome variables, see **S3 Table**). Geographic diversity was measured as effective Shannon's diversity of continent of isolate collection, excluding isolates for which location of collection was unknown. Nucleotide divergence was measured as the median pairwise nucleotide divergence after removal of recombinant regions detected by Gubbins (see **Methods**). **S3 Table** lists the uncorrected p-values for all tests and indicates which of these remain significant at the 5% significance threshold after Bonferroni multiple testing correction (for each outcome variable, n=3 tests).

Only three of the statistical tests were significant after multiple testing correction: Sample size was significantly associated with absolute number of both K and O loci, but not with K or O locus diversity. Sample size was also significantly associated with plasmid replicon diversity. Thus, in order to check that the association between plasmid replicon diversity and clone type described in the main text was not merely an artefact of differing sample sizes, we tested both variables in a combined model (ANCOVA), which indicated that both sample size and clone type were significantly associated with plasmid replicon diversity ($p<0.023$ for all coefficients). The coefficient estimates for the unassigned and MDR clone types were 6.02 and 6.05, respectively ($p=0.0005$ and $p=0.0019$, respectively), supporting greater diversity in the unassigned and MDR clones compared to the hypervirulent clones, even after accounting for differences in sample size.

CG25 as an outlier among the hypervirulent clones
CG25 was identified as an outlier among the hypervirulent clones in terms of the number of genomes harbouring acquired resistance genes (**Figure 1**) and the level of recombination detected (**Figure 2**). Consistent with these observations, comparison of pairwise total gene Jaccard distance distributions showed that CG25 also harboured more gene content diversity than any other hypervirulent clone ($p< 1x10^{-5}$ for each Wilcoxon Rank Sum test). This observation was further supported by comparison of distance to group centroid distributions for all hypervirulent clones except CG65 ($p<0.015$, except CG65 for which $p=0.0878$). CG25 also had the highest plasmid diversity with replicon effective Shannon diversity 7.9 (CG25) vs <6 (all other hypervirulent clones), but no significant difference in plasmid load ($p=0.9346$). Hence the data suggest a higher rate of plasmid turnover (including AMR plasmids) compared to the other hypervirulent clones. Comparisons of the distributions of Euclidean distances from clone centroids for the phage PCA showed that CG25 differed significantly from CG23 ($p=0.0031$), CG66 ($p=0.0218$), CG86 ($p=0.0333$) and CG65 ($p=0.0338$) but not CG380 ($p=0.2178$). Note that only CG23 remained significantly different when multiple testing correction was applied (n=5 tests).

Distribution of CRISPR/Cas systems
Two distinct type 1E *cas* operons have been identified in *K. pneumoniae* [1] and *in vitro* CRISPR activity was shown to limit uptake and maintenance of an MDR plasmid by a hypervirulent CG23 reference strain [2]. We used the CRISPR recognition tool [3] to search for CRISPR arrays and HMM domain profiles [4] to search for the associated *cas* genes. Overall, 456 of 1092 genomes from 26 clones, contained at least one CRISPR array. However, only 344 genomes from 14 clones harboured any of the associated *cas* genes (**S2 Table**). The latter could be divided into two distinct types, matching those described previously for *K. pneumoniae* CG23 strain NTUH-K2044 [2] and CG66 strain Kp52.145 [1]. There appeared to be an association between *cas* type and the number of CRISPR arrays; 181/226 (80.0%) genomes harbouring a partial or complete set of NTUH-K2044-like *cas* genes harboured two CRISPR arrays, 90/118 (76.2%) genomes harbouring a partial or

complete set of Kp52.145-like *cas* genes harboured only one CRISPR array (**S2 Table, S10 Fig**). We used the combination of CRISPR array and *cas* gene data to identify genomes with putative functional CRISPR/Cas systems (i.e. containing at least one CRISPR array plus a complete set of 8 *cas* genes, n=324 genomes). There were 9 clones for which the majority of genomes (≥75%) harboured a putative functional system (one hypervirulent, two MDR, 6 unassigned). A further four clones included a minority of genomes with putative functional systems (7-50% genomes, **S10 Fig**). Both *cas* operons were represented across all clone types. Four of the 6 hypervirulent clones contained no intact CRISPR-Cas loci and two MDR clones (CG147 and CG15) showed high conservation of putative intact loci despite evidence of frequent recombination and high plasmid diversity (though we cannot comment on the relative activity of these systems, which may be subject to distinct regulatory controls). Hence while CRISPR/Cas may contribute to limiting recombination and plasmid acquisition in two hypervirulent clones within which these systems were identified (CG23 and CG66), it is not a general barrier to DNA uptake in all hypervirulent clones.

Distribution of R-M systems
We also explored the potential impact of R-M systems. We used HMM profiles[5,6] to search for R-M restriction enzymes (REases) among all 1092 genomes in the 28 common clones, and identified 33, 13, 7 and 13 distinct clusters of Types I, II, III and IV REases, respectively. We defined clusters at the amino acid identity levels considered appropriate to distinguish enzymes targeting distinct nucleotide sequence motifs (see **Methods** and [5]). Overall 542, 374, 303 and 197 genomes harboured ≥1 distinct type I, II, III and IV REase genes, respectively. The total number of distinct REases differed between groups such that compared to the hypervirulent genomes, MDR genomes tended to harbour greater numbers of distinct REases of each type (**S11 Fig, S2 Table,** $p \leq 0.0031$ for all pairwise Wilcoxon Rank Sum tests). MDR clones also seemed to be associated with greater REase gene diversity (**S12 Fig**), a finding consistent with a previous report showing bacterial species subject to higher rates of horizontal gene transfer were associated with greater REase diversity [5]. Next we sought to explore whether the distribution of REase clusters resulted in differences in the potential to receive incoming DNA. To do this we searched a diverse collection of 1722 *K. pneumoniae* genomes for representatives of each REase cluster, and determined the potential compatibility of each common clone genome as a recipient of DNA from each genome in the broader collection (the donors, see **Methods**). The distributions of compatible pairings suggest differences between the three groups of clones (pairwise Wilcoxon Rank Sum tests; MDR vs hypervirulent, $p < 1x10^{-13}$; unassigned vs hypervirulent, $p < 1x10^{-6}$; unassigned vs MDR, $p < 0.001$), with lower compatibility among the MDR clones consistent with the greater number and diversity of REase genes in these genomes. Hence, these data do not support a major role for R-M systems in reducing DNA uptake in hypervirulent clones beyond that of the unassigned or MDR clones (**S13 Fig)**. However it is important to note that these analyses are subject to two key caveats; 1) assumption that genomes carrying a particular REase also carry the corresponding MTase, ignoring orphan MTases, which are known to exist in bacterial populations [5] and when present in a donor should increase its pool of compatible recipients; 2) clustering of REases on the basis of sequence similarity rather than demonstrated target-site specificities- while the latter is considered a suitable approximation of the former [5], we cannot rule out the possibility that a minority of REases were clustered inappropriately. Nevertheless, our analyses provided a good first estimate of the potential impact of R-M systems in the *K. pneumoniae* population and did not support R-M as a general limiting factor for DNA uptake in hypervirulent clones. Rather our findings suggest that R-M systems may have a greater impact on the MDR clones.

Impact of the K2 capsule on CG15 recombination and pan-genome diversity
In the absence of general differences in DNA defence mechanisms, we hypothesised that the hypervirulent clones may be physically inhibited from DNA uptake by overexpression of their capsules, in particular the thick K2 capsule that dominated five of the 6 hypervirulent clones in our analyses. If this were indeed the case, we would expect that presence of the corresponding KL2 locus among a subset of genomes from an MDR or unassigned clone would also be associated with a comparative reduction in chromosomal recombination and pan-genome diversity. In the current study collection KL2 was identified among four MDR/unassigned clones, including one with sufficient genome numbers for meaningful comparison (CG15,

with 31 KL2 genomes and 66 non-KL2 genomes). By overlaying K locus types onto the CG15 recombination-free phylogeny, we see that KL2 is confined to one of two major subclades (**S14 Fig**). Comparison of r/m values indicated that the CG15-KL2 subclade (including 31 KL2 genomes plus a single KL157 genome that clustered within the subclade) has experienced a lower rate of recombination than the other major subclade (associated with extensive K locus diversity, labelled CG15-other in **S14 Fig,** r/m; 0.58 vs 6.75). In addition, the CG15-KL2 subclade was associated with reduced pan-genome diversity compared to CG15-other (median pairwise Jaccard gene distance 0.1339 vs 0.1487, Wilcoxon Rank Sums test $p < 1x10^{-7}$; pan-genome curve alpha values 0.785 vs 0.686; see **S14 Fig**). However, this was not supported by a comparison of pan-genome distance to group centroids (p=0.2451).

To estimate the length of time over which CG15-KL2 has maintained the KL2 locus we performed a BEAST2 [7] analysis on the subset of genomes for which isolate collection years were known (n=21, spanning 2002–2013, see **S2 Table**). The evolutionary rate was estimated as $2.59x10^{-6}$ substitutions $site^{-1}$ $year^{-1}$ (95% Highest Posterior Density (HPD): $2.16x10^{-6}$ - $3.27x10^{-6}$), slightly faster than that estimated previously for ST258 (95% HPD: $8.09x10^{-7}$ - $1.24x10^{-6}$) [8] and ST307 (95% HPD: $8.01x10^{-7}$–$1.58x10^{-6}$) [9], which in turn were faster than our recent estimate for CG23 (95% HPD: $2.43x10^{-7}$ - $4.38x10^{-7}$) [10]. The estimated date of the most recent common ancestor for CG15-KL2 was 1979 (95% HPD: 1974 - 1980), suggesting that the KL2 locus has been maintained for at least 34 years prior to the most recent isolate in the analysis (collected in 2013), with only a single change of K locus detected in this time (**S14 Fig**).

References

1. Shen J, Lv L, Wang X, Xiu Z, Chen G. Comparative analysis of CRISPR-Cas systems in *Klebsiella* genomes. J Basic Microbiol. 2017; 1–12. doi:10.1002/jobm.201600589
2. Lin T-L, Pan Y-J, Hsieh P-F, Hsu C-R, Wu M-C, Wang J-T. Imipenem represses CRISPR-Cas interference of DNA acquisition through H-NS stimulation in *Klebsiella pneumoniae*. Sci Rep. 2016;6: 31644. doi:10.1038/srep31644
3. Bland C, Ramsey TL, Sabree F, Lowe M, Brown K, Kyrpides NC, et al. CRISPR recognition tool (CRT): a tool for automatic detection of clustered regularly interspaced palindromic repeats. BMC Bioinformatics. 2007;8: 209. doi:10.1186/1471-2105-8-209
4. Burstein D, Harrington LB, Strutt SC, Probst AJ, Anantharaman K, Thomas BC, et al. New CRISPR–Cas systems from uncultivated microbes. 2017;542: 237–241. doi:10.1038/nature21059
5. Oliveira PH, Touchon M, Rocha EPC. Regulation of genetic flux between bacteria by restriction–modification systems. Proc Natl Acad Sci U S A. 2016;113: 5658–5663. doi:10.1073/pnas.1603257113
6. Cury J, Jové T, Touchon M, Néron B, Rocha EP. Identification and analysis of integrons and cassette arrays in bacterial genomes. Nucleic Acids Res. 2016;44: 4539–4550. doi:10.1093/nar/gkw319
7. Bouckaert R, Heled J, Kühnert D, Vaughan T, Wu CH, Xie D, et al. BEAST 2: A software platform for Bayesian evolutionary analysis. PLoS Comp Biol. 2014;10: e1003537. doi:10.1371/journal.pcbi.1003537
8. Bowers JR, Kitchel B, Driebe EM, MacCannell DR, Roe C, Lemmer D, et al. Genomic analysis of the emergence and rapid global dissemination of the clonal group 258 *Klebsiella pneumoniae* pandemic. PLoS One. 2015;10: e0133727. doi:10.1371/journal.pone.0133727
9. Wyres KL, Hawkey J, Hetland MAK, Fostervold A, Wick R, Judd LM, et al. Emergence and rapid global dissemination of CTX--M--15-associated *Klebsiella pneumoniae* strain ST307. J Antimicrob Chemother. 2019;74: 577–581.
10. Lam MMC, Wyres KL, Duchêne S, Wick RR, Judd LM, Gan Y, et al. Population genomics of hypervirulent *Klebsiella pneumoniae* clonal group 23 reveals early emergence and rapid

global dissemination. Nat Commun. 2018;9: 2703.
