# Supplementary figures and images for: Distinct evolutionary dynamics of horizontal gene transfer in drug resistant and virulent clones of Klebsiella pneumoniae
Source: PLoS Genet. 2019 Apr 15;15(4):e1008114. doi: 10.1371/journal.pgen.1008114 (PMC6483277; doi:10.1371/journal.pgen.1008114)

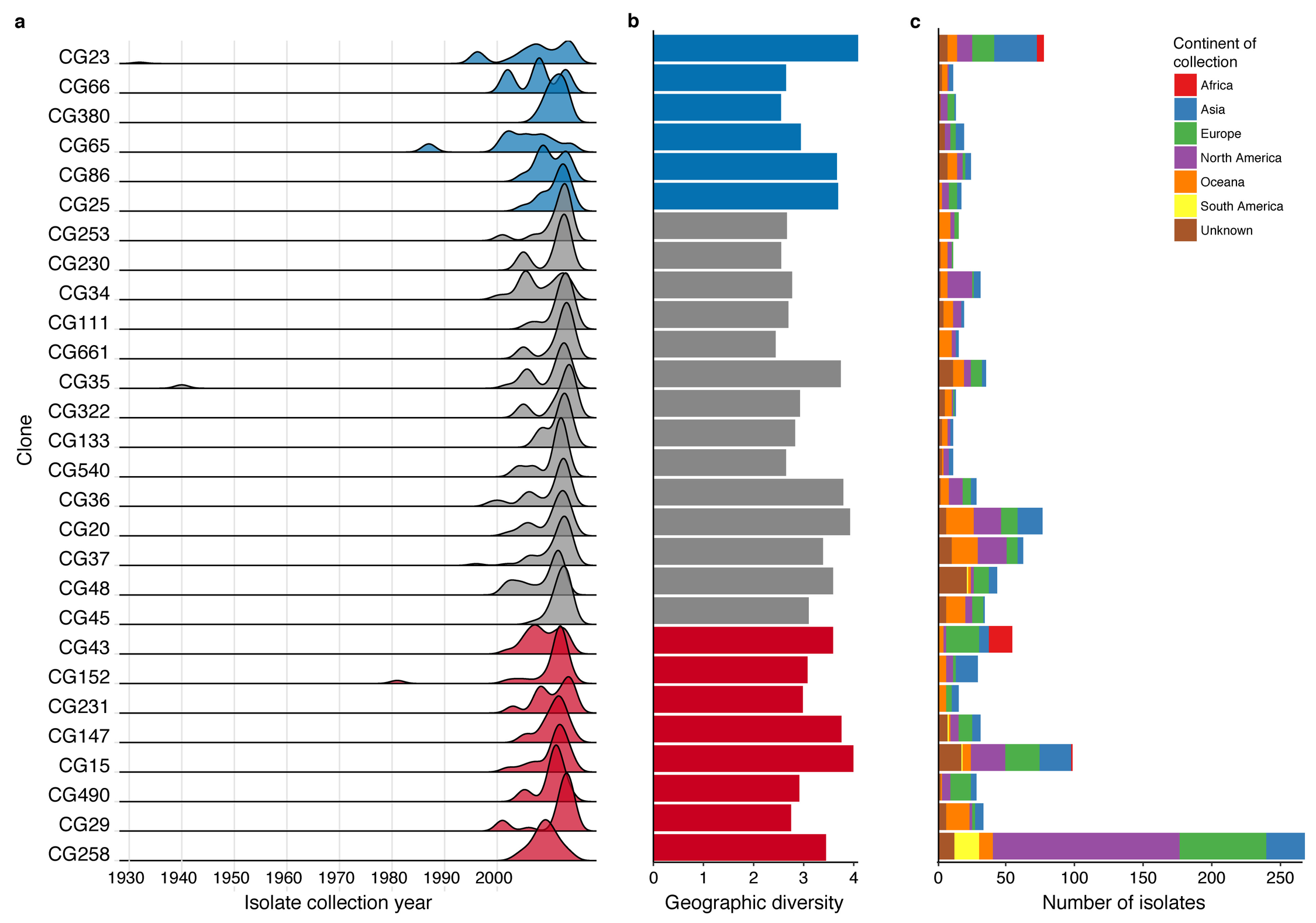

Supplement: S1 Fig — a) Density plots showing the distribution of years of collection for all isolates for which years were known (see S1 and S2 Tables). Distributions are coloured by clone type; blue, hypervirulent; grey, unassigned; red, multi-drug resistant. b) Effective Shannon’s diversity of continent of collection. Bars are coloured by clone type as in (a). c) Count of isolates represented in each clone, coloured by continent of collection as indicated. (TIF) [file pgen.1008114.s005.tif]

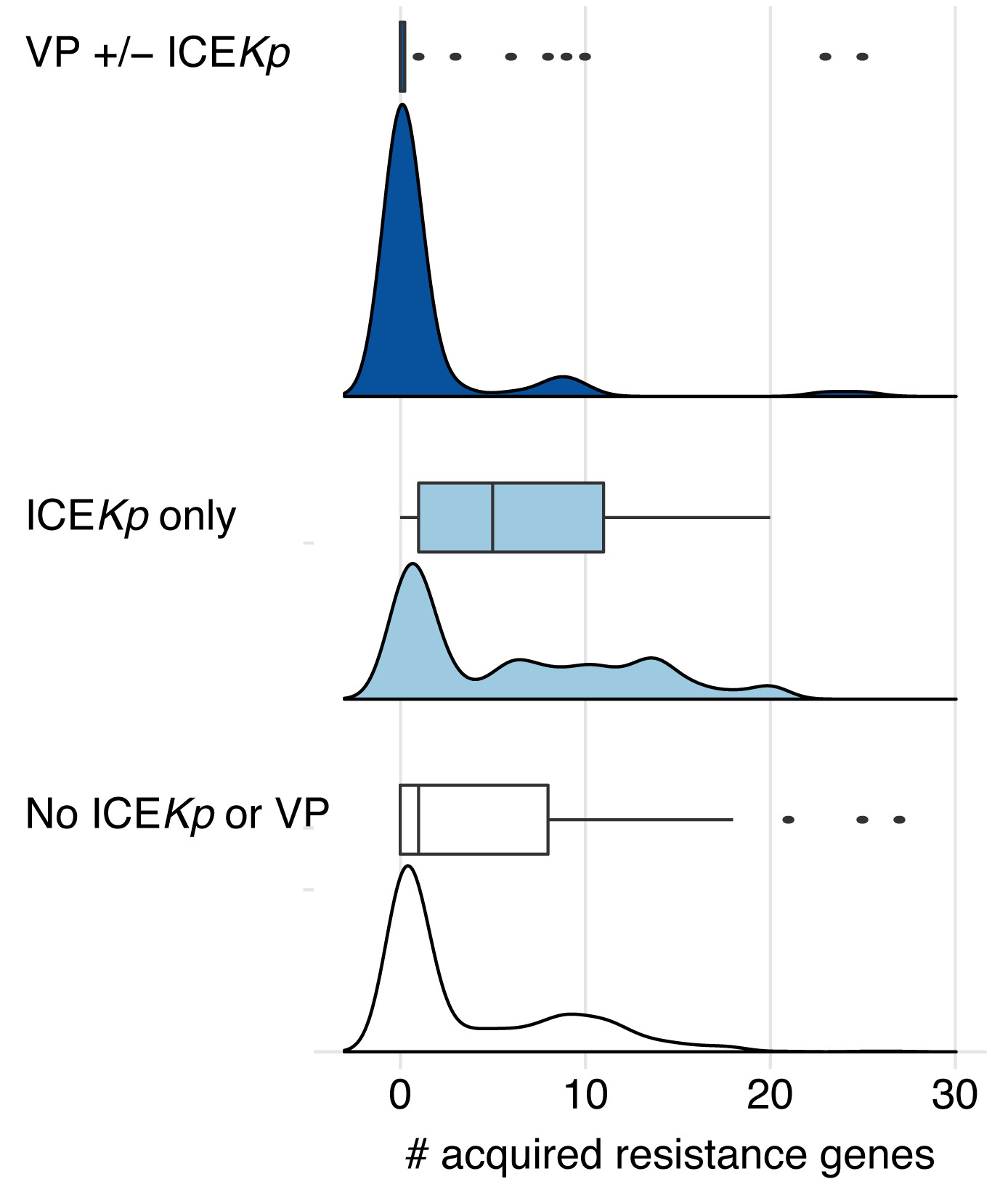

Supplement: S2 Fig — Density plots and matched boxplots show data for 1124 genomes that can be considered representative of the broader population of genomes (i.e. with isolates from known outbreaks and overrepresented clones subsampled, as described previously in Wyres et al MGen 2016). Data are stratified by virulence status, defined as follows: No ICEKp or VP = none of the yersiniabactin, colibactin or aerobactin loci were identified, n = 781 (the former two are indicative of the ICEKp and the latter is indicative of the virulence plasmid, VP); ICEKp only = the yersiniabactin synthesis locus plus/minus the colibactin synthesis locus was identified without the aerobactin locus, n = 255; VP +/- ICEKp = the aerobactin locus was identified plus/minus the yersiniabactin and/or colibactin loci, n = 88. (TIF) [file pgen.1008114.s006.tif]

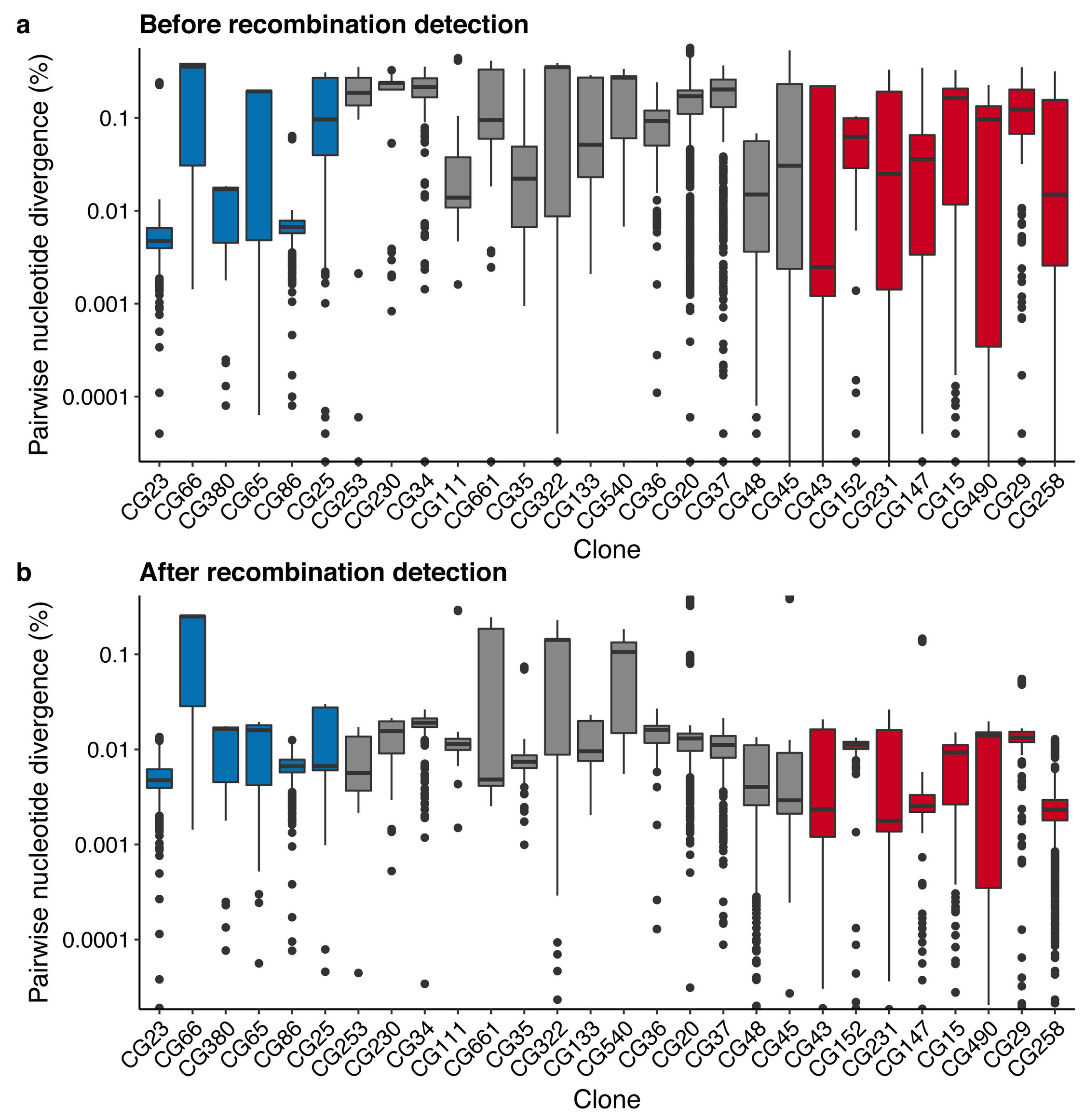

Supplement: S3 Fig — Distributions of pairwise nucleotide divergence before (a) and after (b) removal of recombinant sequence regions. Boxplots are coloured by clone type; blue, hypervirulent; grey, unassigned; red, multi-drug resistant. (TIF) [file pgen.1008114.s007.tif]

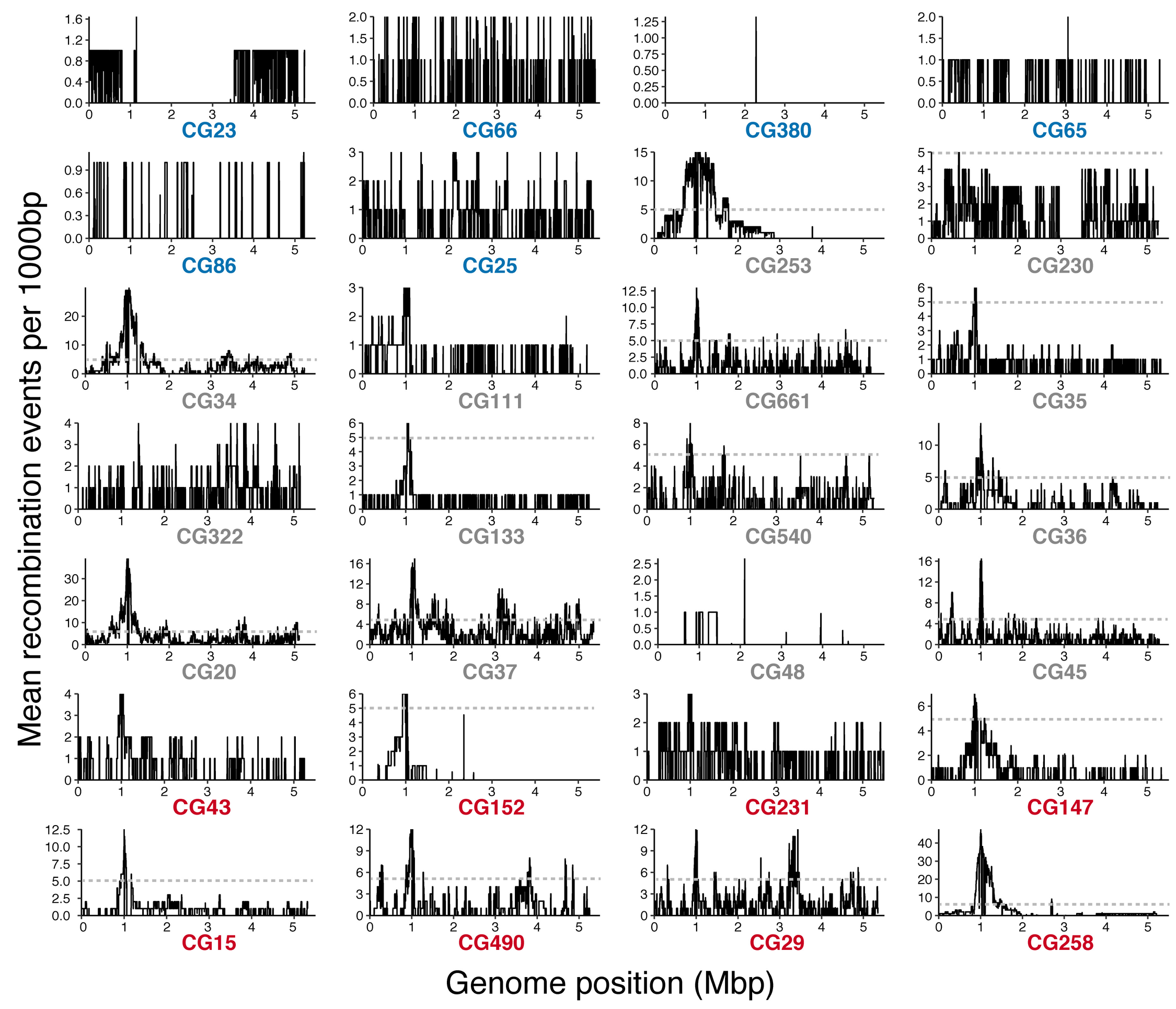

Supplement: S4 Fig — Mean recombination events per base (calculated across non-overlapping 1000 bp windows, relative to the clone reference chromosome—see S1 Table) are shown for each clone, ordered from left-to-right by row as in Fig 1B and 1C. Y-axes represent mean counts (note that scales differ), and x-axes represent genome position relative to the reference genome for the given clone (coordinates in Mbp). Chromosomes are aligned such that the galF K locus gene starts at 1 Mbp. Grey dashed lines indicate mean = 5 recombination events. Clone labels are coloured by clone type; blue, hypervirulent; grey, unassigned; red, multi-drug resistant. (TIF) [file pgen.1008114.s008.tif]

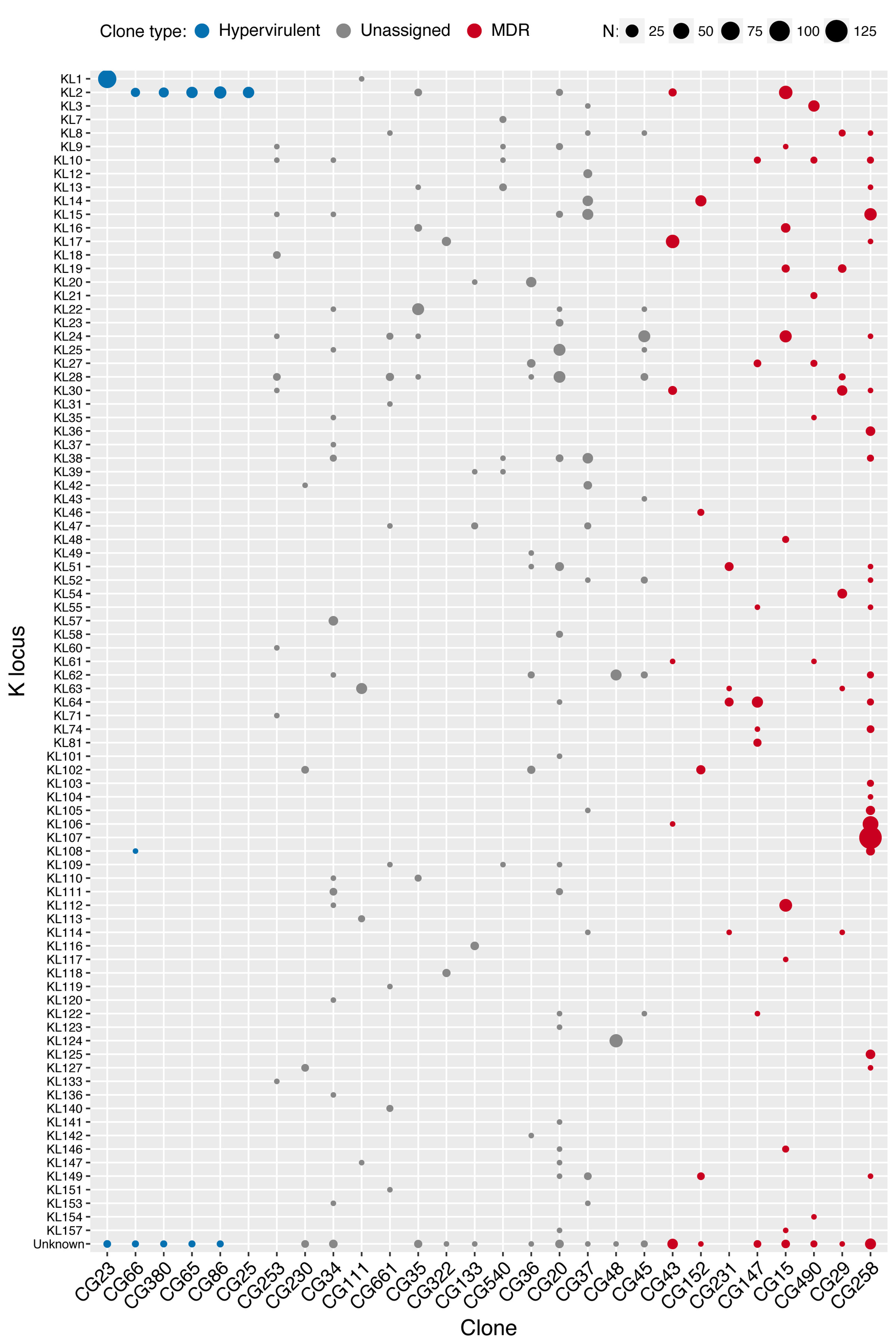

Supplement: S5 Fig — K locus assignments for which the Kaptive match confidence was “Good” or better are shown. Low or no confidence matches were grouped as “Unknown.” Data points are scaled proportional to the number of genomes they represent and coloured by clone type as indicated. (TIF) [file pgen.1008114.s009.tif]

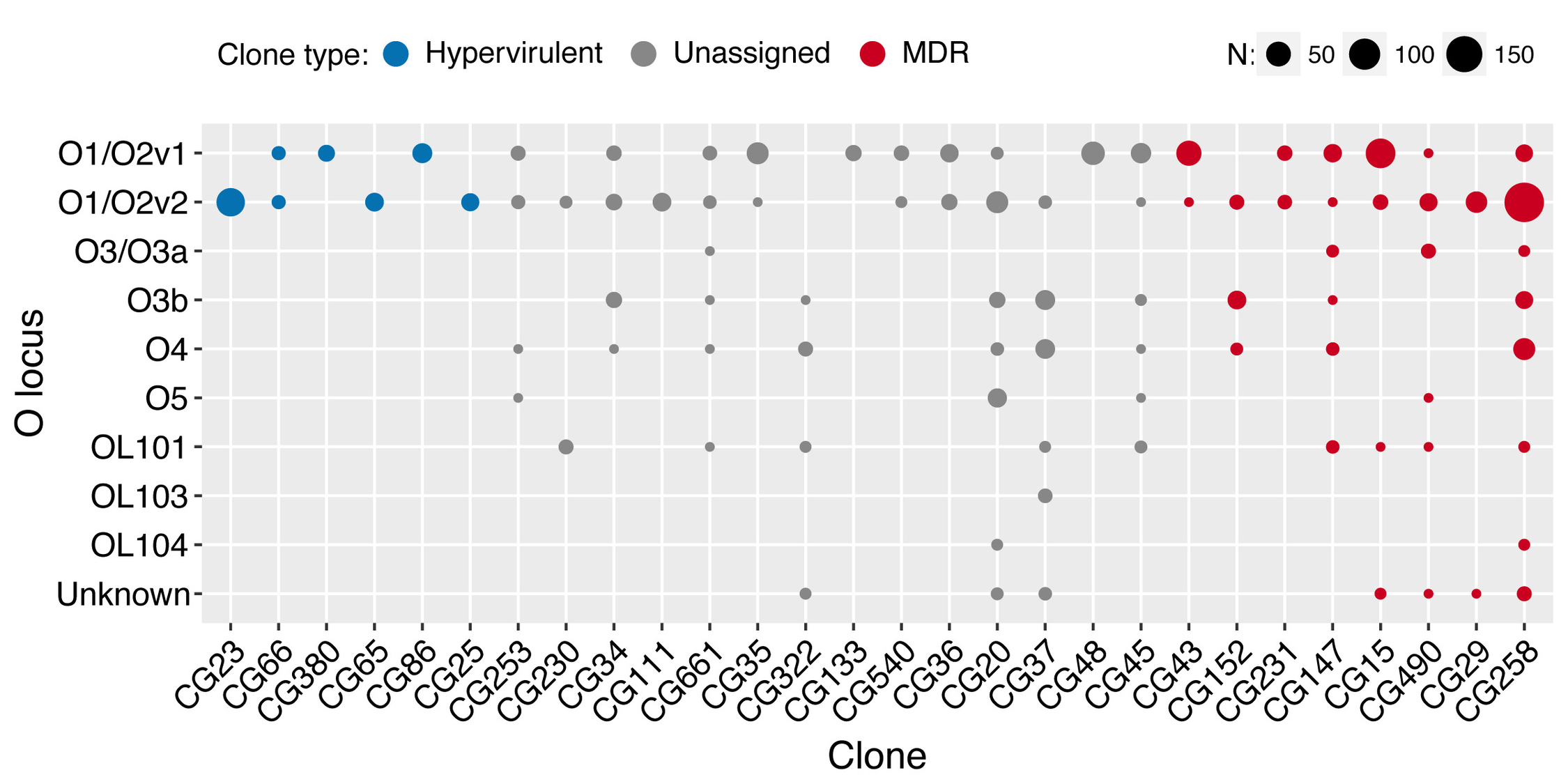

Supplement: S6 Fig — O locus assignments for which the Kaptive match confidence was “Good” or better are shown. Low or no confidence matches were grouped as “Unknown.” Data points are scaled proportional to the number of genomes they represent and coloured by clone type as indicated. The O1 and O2 lipopolysaccharides are both associated with two distinct O locus variants (v1 and v2). Distinction between the O1 and O2 phenotypes is dependent on the presence of genes located elsewhere in the genome (not shown). (TIF) [file pgen.1008114.s010.tif]

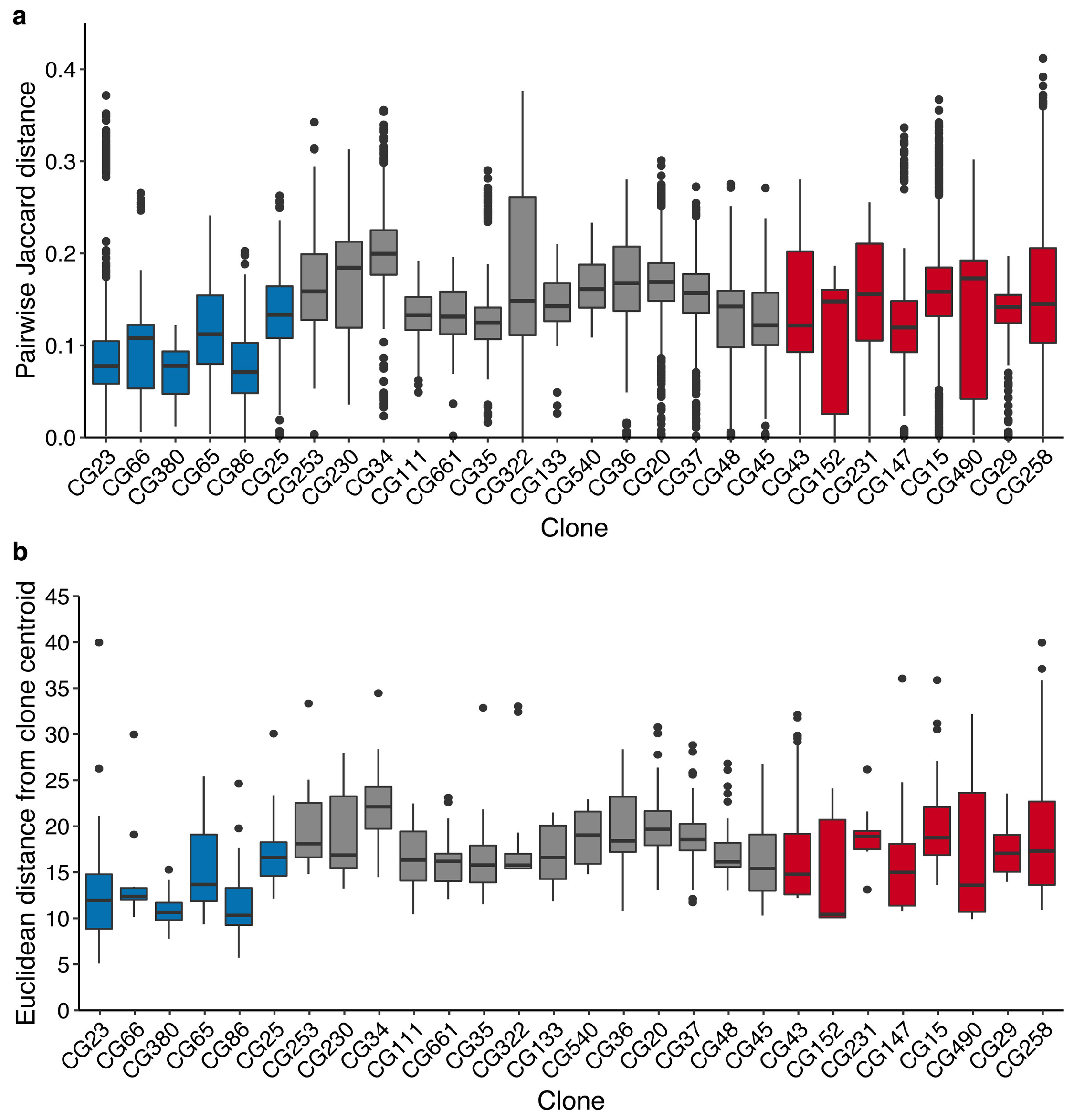

Supplement: S7 Fig — a) Jaccard gene distances. Data points within each distribution represent pairwise comparisons for each pair of genomes within the clone. b) Euclidean distances from clone centroids calculated from the pan-genome gene content matrix after decomposition to 463 dimensions (see Methods). Data points within each distribution represent single genomes. In both panels boxplots are coloured by clone type; blue, hypervirulent; grey, unassigned; red, multi-drug resistant. (TIF) [file pgen.1008114.s011.tif]

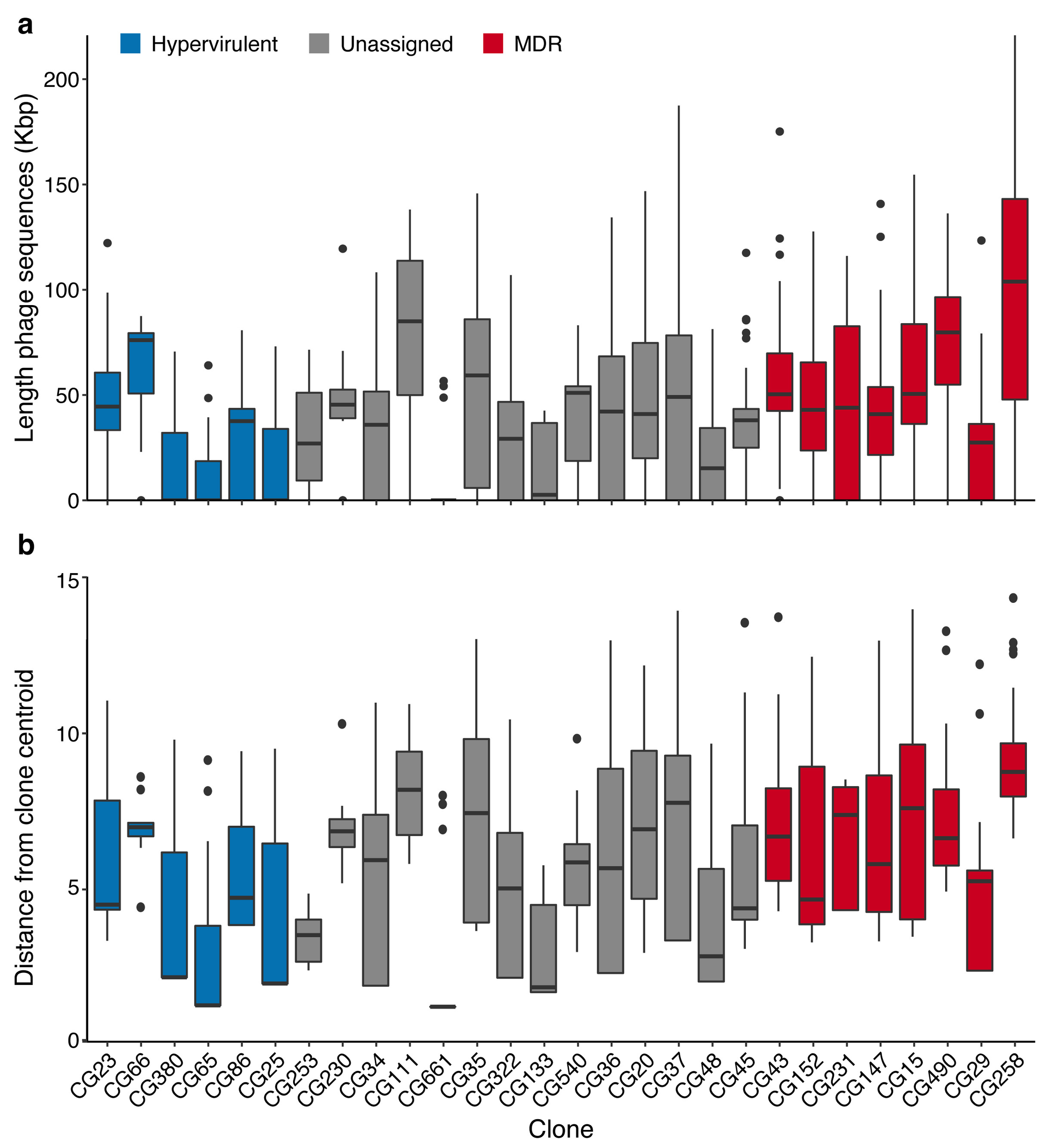

Supplement: S8 Fig — a) Boxplots show the distributions of the total length (kbp) of phage sequence identified per genome. b) Boxplots show the distributions of Euclidean distance to clone centroids calculated from the phage gene presence matrix decomposed into 210 dimensions. (TIF) [file pgen.1008114.s012.tif]

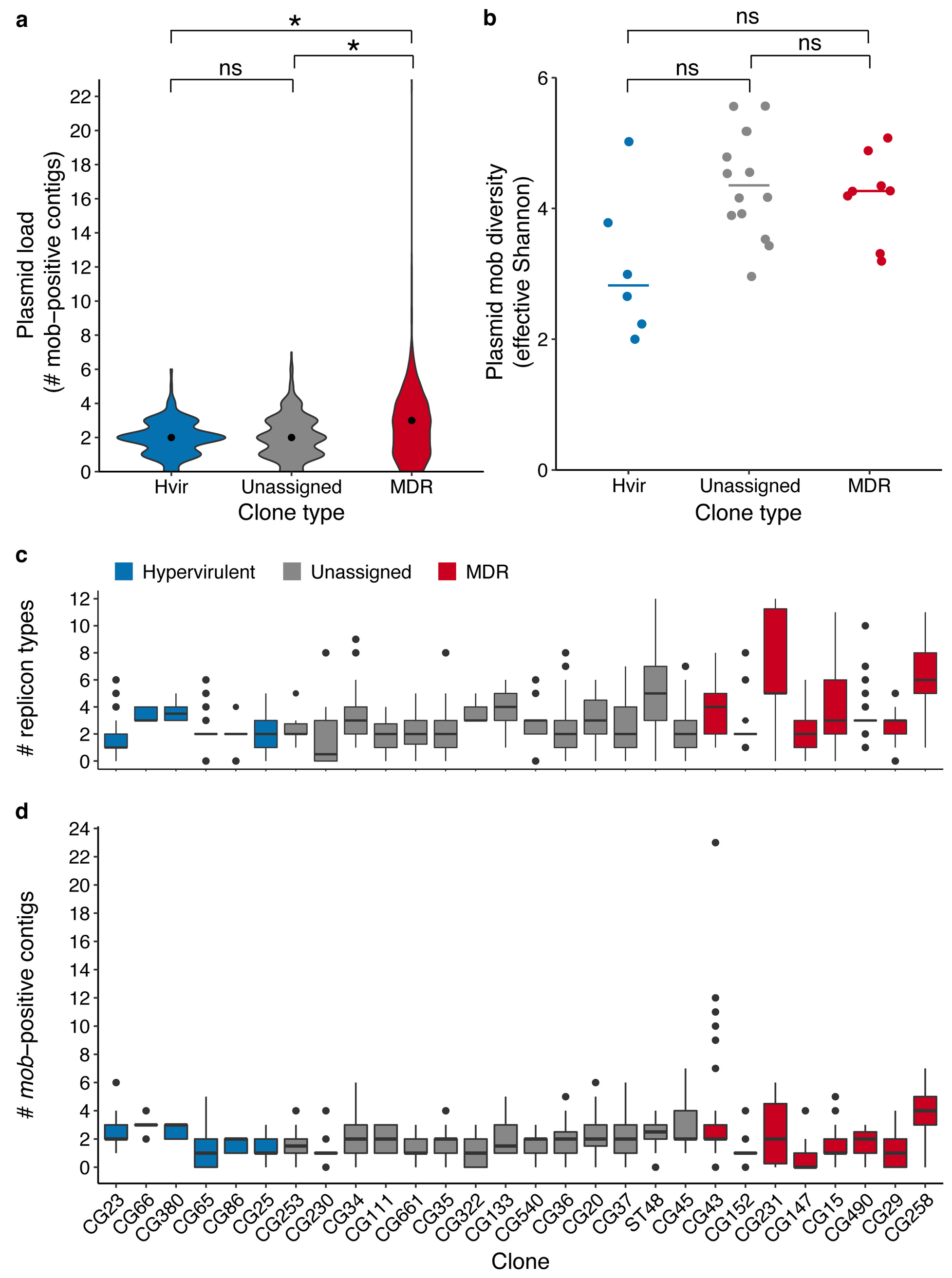

Supplement: S9 Fig — a) Distributions of number of mob-positive contigs by clone type. Each data point represents a single genome, grouped by clone type. b) Effective Shannon’s diversity of mob types. Each data point represents a single clone grouped by clone type. c) Counts of unique plasmid replicon types (perfectly co-occurring types counted once only). d) Counts of mob-positive genome assembly contigs. Each data point in (c) and (d) represents a single genome. Clones are coloured by clone type as indicated. For panels (a) and (b), brackets indicate Wilcoxon Rank Sum tests of pairwise group comparisons; ns, not significant; *, p < 0.01. (TIF) [file pgen.1008114.s013.tif]

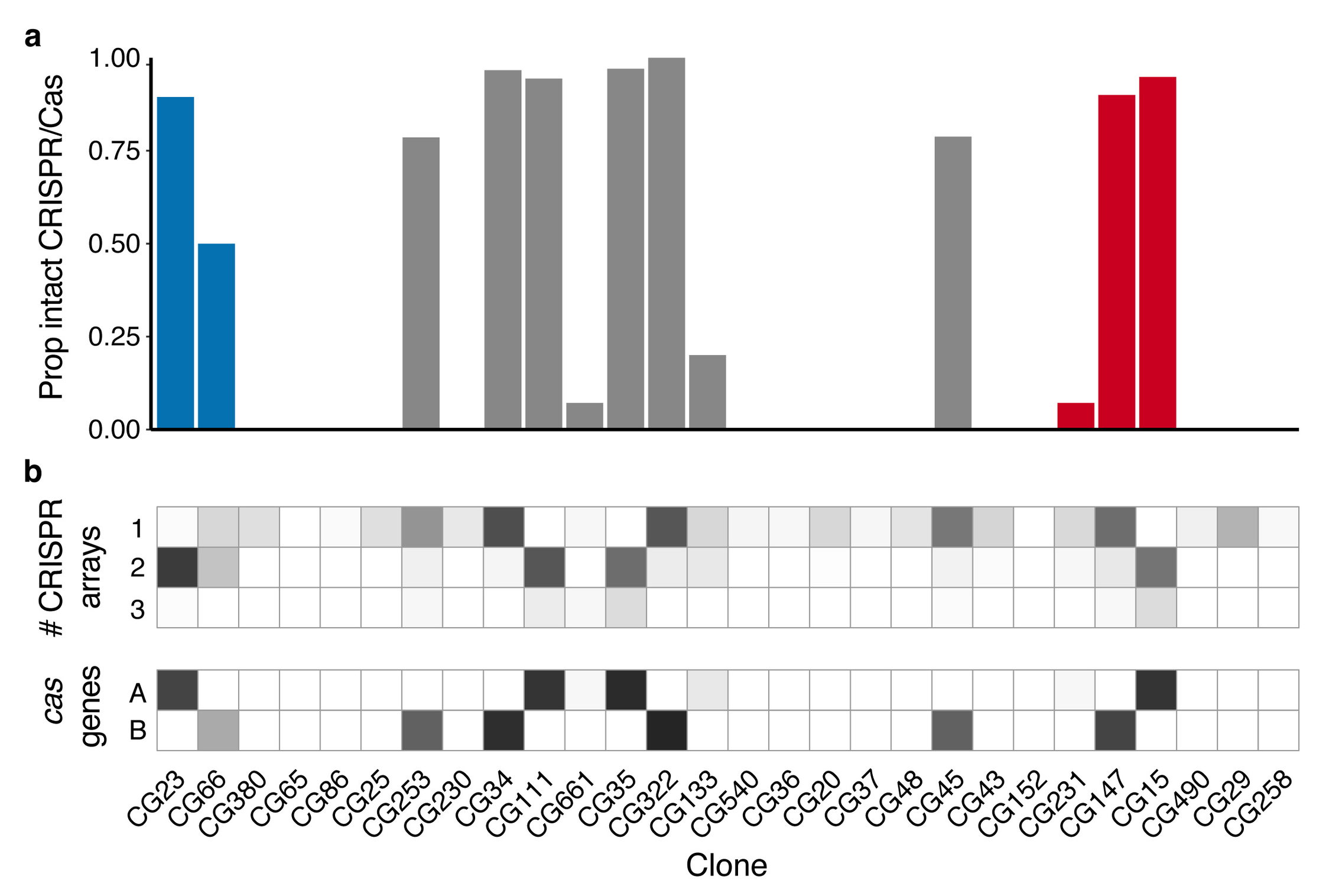

Supplement: S10 Fig — a) Proportion of genomes harbouring putative intact CRISPR/Cas loci i.e. those harbouring at least one CRISPR array and a complete set of 8 cas genes. Bars are coloured by clone type; blue, hypervirulent; grey, unassigned; red, multi-drug resistant. b) Proportion of genomes harbouring 1, 2, or 3 CRISPR arrays (upper) and proportion of genomes harbouring at least one cas gene of types A (NTUH-K2044-like) or B (Kp52.145-like). White, 0 genomes; dark grey, all genomes. (TIF) [file pgen.1008114.s014.tif]

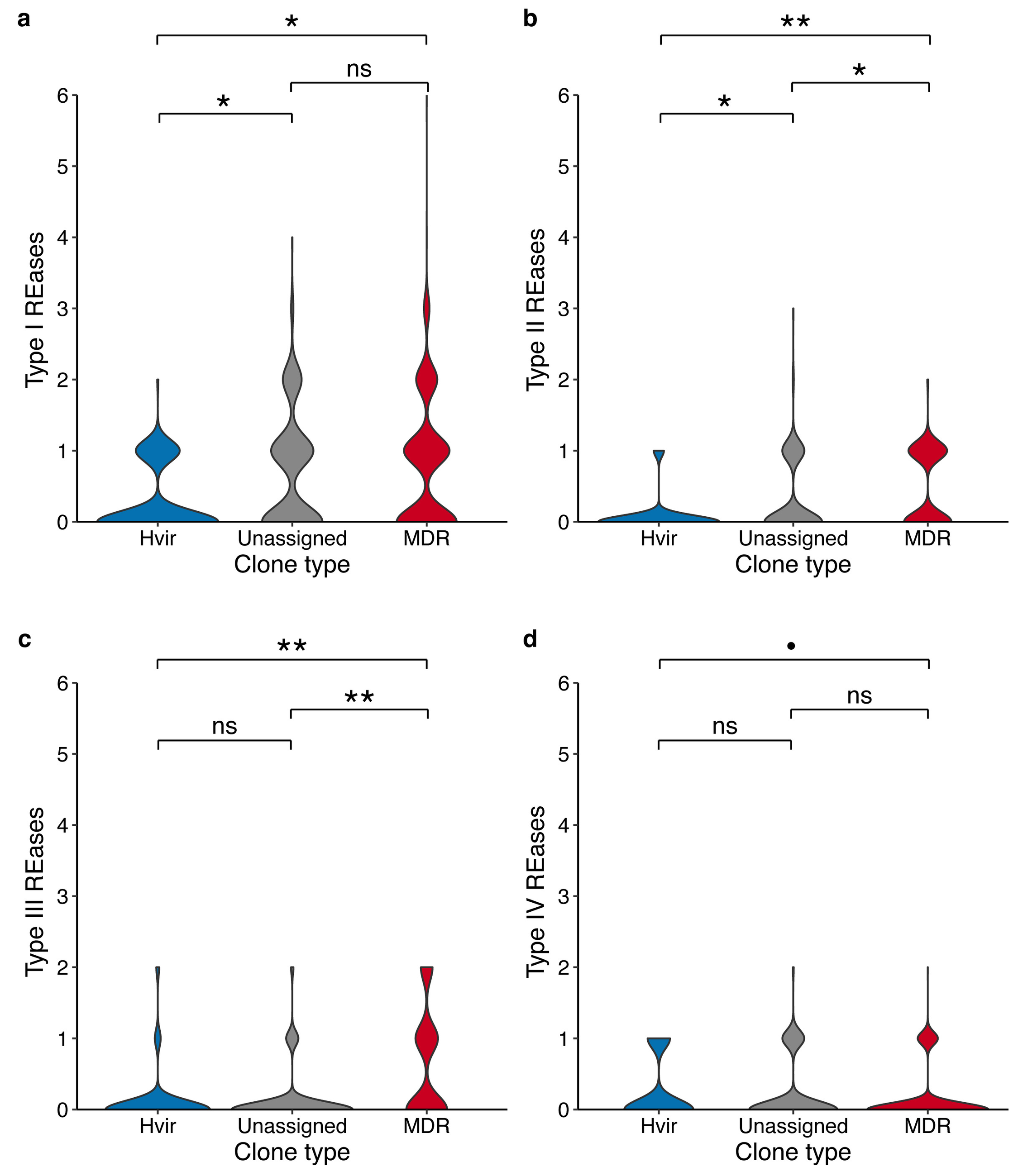

Supplement: S11 Fig — Violin plots show the distributions of the number of distinct REases identified in each genome; a) Type I REases; b) Type II REases; c) Type III REases; d) Type IV REases. Brackets indicate Wilcoxon Rank Sum tests for pairwise comparisons; ns, not significant after Bonferroni multiple testing correction; ▭, p < 0.0042; *, p < 0.001; **, p < 1x10^-15^. (TIF) [file pgen.1008114.s015.tif]

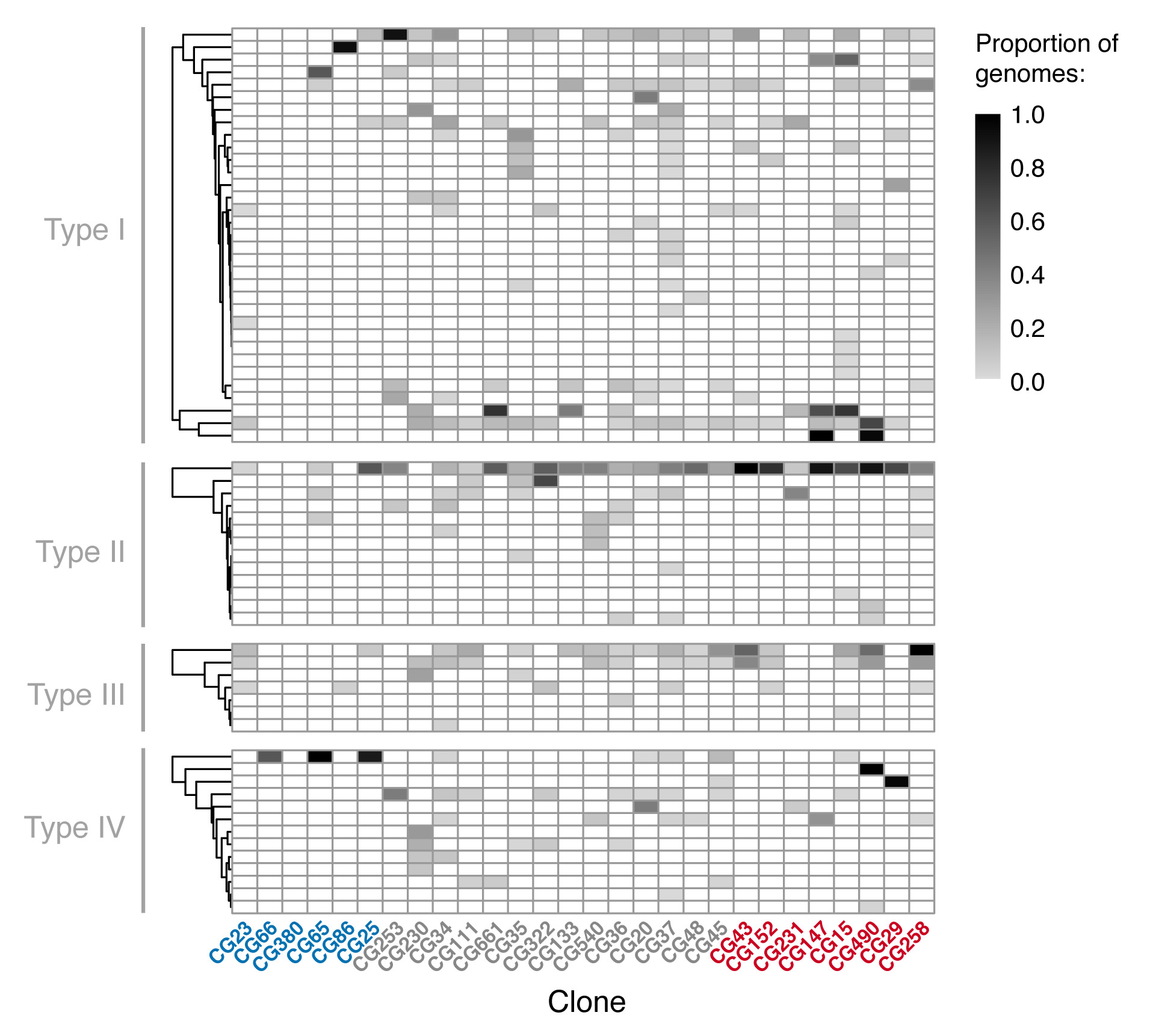

Supplement: S12 Fig — Heatmap showing the proportion of genomes for each clone that were positive for ≥1 copy of each of 33 type I, 13 type II, 7 type III and 13 type IV REase genes (shown in rows). Clone names are coloured by clone type; blue = hypervirulent; grey = unassigned; red = multi-drug resistant. (TIF) [file pgen.1008114.s016.tif]

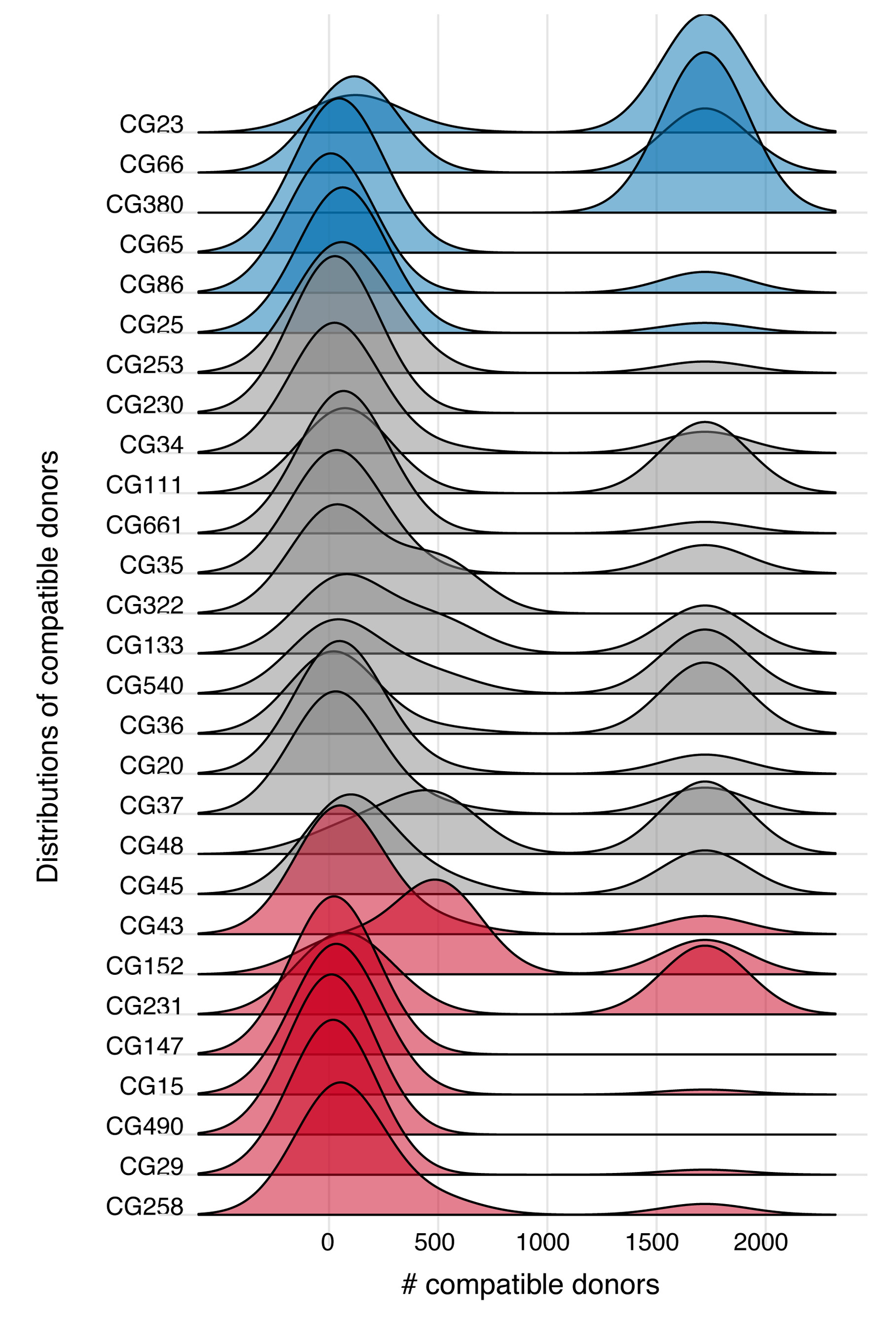

Supplement: S13 Fig — Data points represent individual genomes and show the number of compatible donors from a collection of 1722 diverse K. pneumoniae. (TIF) [file pgen.1008114.s017.tif]

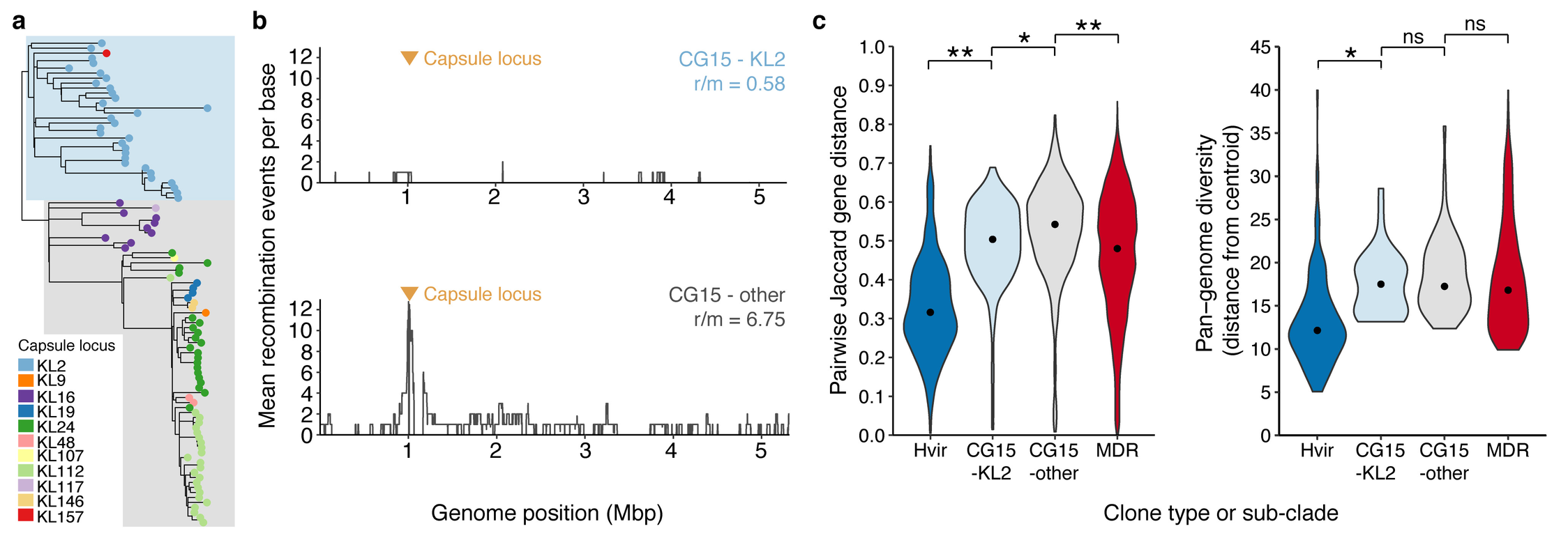

Supplement: S14 Fig — a) Recombination-free maximum-likelihood phylogeny (mid-point rooted) with tips coloured by capsule (K) locus as indicated. The CG15-KL2 (blue) and CG15-other (grey, diverse K loci) subclades are marked. The divergence date for the CG15-KL2 subclade, estimated by BEAST2 analysis of the 21 CG15-KL2 genomes with known isolation dates, was estimated to be 1970 (95% HPD, 1974–1980). b) Recombination events within the CG15-KL2 and CG15-other subclades. Mean recombination events per base calculated over non-overlapping 1000 bp windows of the chromosome are plotted by position in the CG15 reference genome PMK1 (accession: CP008929) adjusted such that the capsule locus is shown at 1 Mbp. c) and d) Pan-genome diversity of the CG15-KL2 and CG15-other subclades compared to the hypervirulent clones and the remaining MDR clones. c) Violin plots show the distribution of pairwise Jaccard gene distances. d) Violin plots show the distributions of Euclidean distances from clone or subclade centroids, calculated from pan-genome gene content matrix after decomposition to 463 dimensions as in Fig 4C. Brackets indicate Wilcoxon Rank Sum tests for pairwise comparisons; ns, not significant; *, p < 1x10^-6^; **, p < 1x10^-15^. (TIF) [file pgen.1008114.s018.tif]

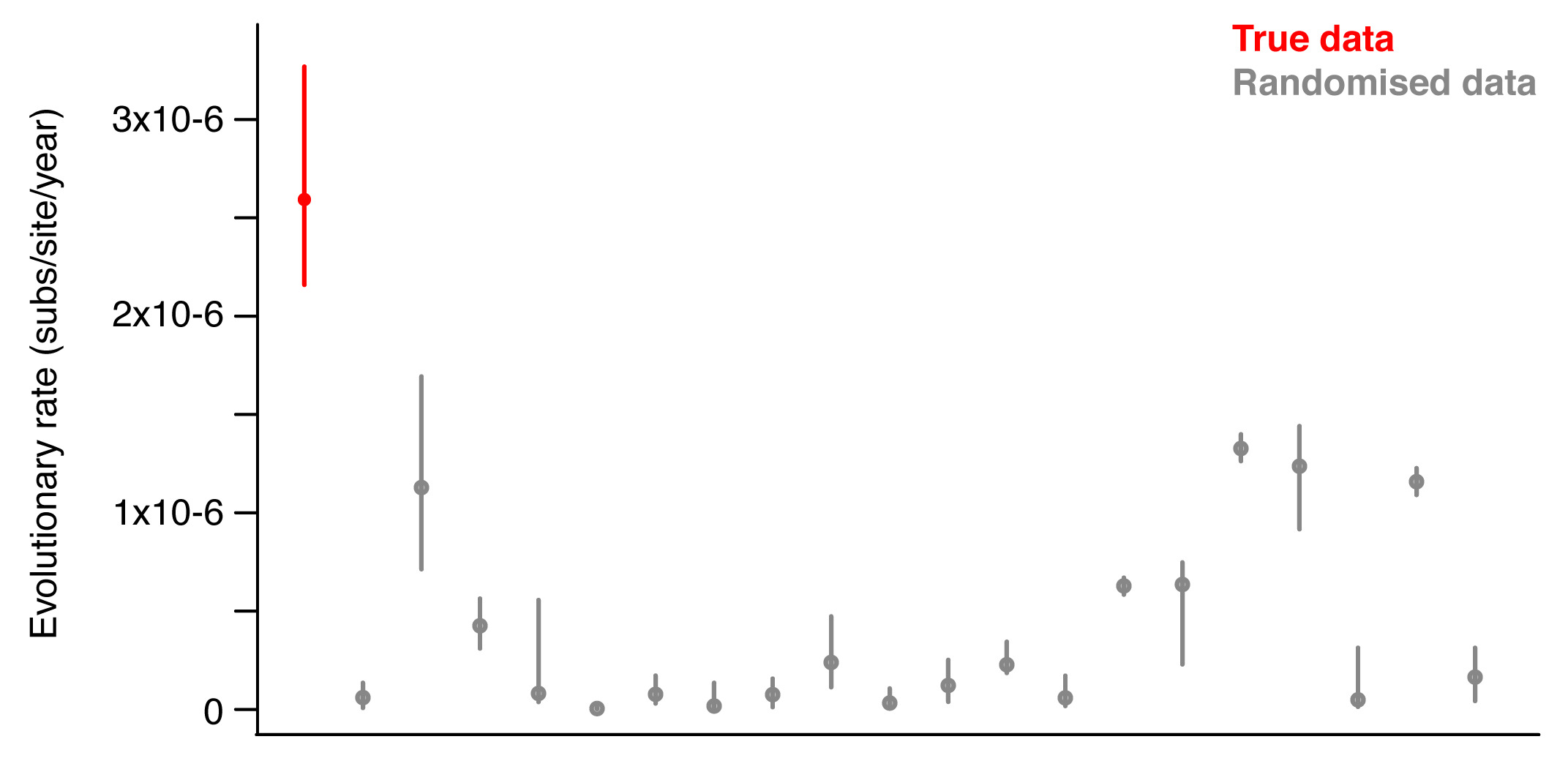

Supplement: S15 Fig — The median estimates and ranges are shown for the true CG15-KL2 data (red) and CG15-KL2 sequence data with 20 independent date randomisations (grey). (TIF) [file pgen.1008114.s019.tif]

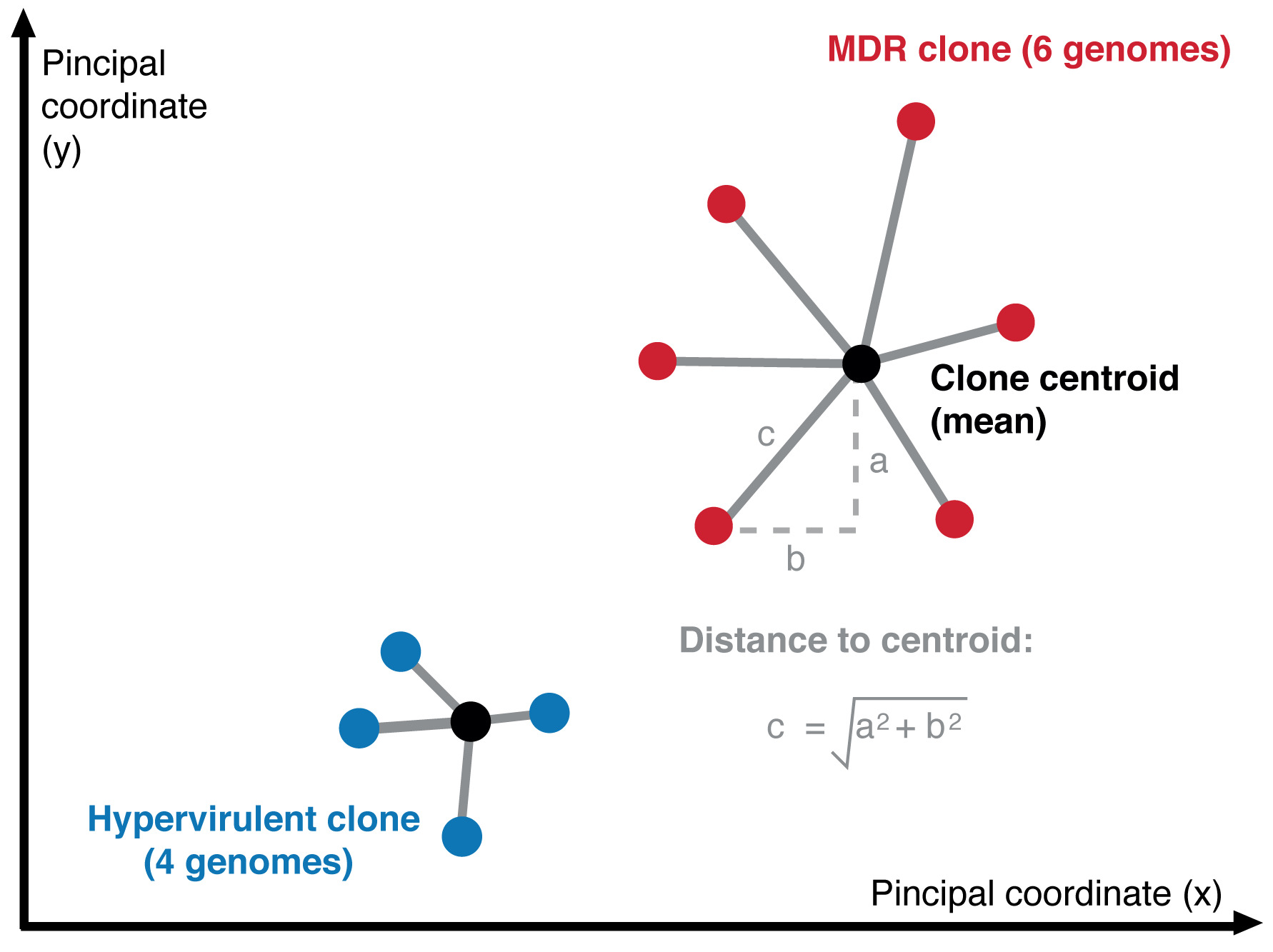

Supplement: S16 Fig — Genomes representing one hypervirulent (blue, n = 4) and one MDR (red, n = 6) clone are shown projected in a 2D space where the position of each genome (x, y) is determined by the relevant values for principal components x and y. Clone centroids are calculated and plotted as the vector of mean coordinates i.e. (μx, μy). For a given genome at position (x1, y1) the distance to clone centroid can be calculated as the Euclidean distance, c: c = √(a2 + b2) = √((μx−x1)2 + (μy−y1)2) (TIF) [file pgen.1008114.s020.tif]
